# Aquatic birds have middle ears adapted to amphibious lifestyles

**DOI:** 10.1038/s41598-022-09090-3

**Published:** 2022-03-28

**Authors:** Jeffrey N. Zeyl, Edward P. Snelling, Maelle Connan, Mathieu Basille, Thomas A. Clay, Rocío Joo, Samantha C. Patrick, Richard A. Phillips, Pierre A. Pistorius, Peter G. Ryan, Albert Snyman, Susana Clusella-Trullas

**Affiliations:** 1 Department of Botany and Zoology, Stellenbosch University, Stellenbosch, South Africa; 2 Department of Anatomy and Physiology, and Centre for Veterinary Wildlife Research, Faculty of Veterinary Science, University of Pretoria, Onderstepoort, South Africa; 3 Marine Apex Predator Research Unit, Department of Zoology, Institute for Coastal and Marine Research, Nelson Mandela University, Port Elizabeth, South Africa; 4 Department of Wildlife Ecology and Conservation, Fort Lauderdale Research and Education Center, University of Florida, Davie, FL USA; 5 School of Environmental Sciences, University of Liverpool, Liverpool, UK; 6 Institute of Marine Sciences, University of California Santa Cruz, Santa Cruz, CA 95064 USA; 7 British Antarctic Survey, Natural Environment Research Council, Cambridge, UK; 8 Department of Biological Sciences, DSI-NRF Centre of Excellence, FitzPatrick Institute of African Ornithology, University of Cape Town, Rondebosch, South Africa; 9 Southern African Foundation for the Conservation of Coastal Birds (SANCCOB), Cape Town, South Africa

**Keywords:** Evolution, Physiology, Zoology

## Abstract

Birds exhibit wide variation in their use of aquatic environments, on a spectrum from entirely terrestrial, through amphibious, to highly aquatic. Although there are limited empirical data on hearing sensitivity of birds underwater, mounting evidence indicates that diving birds detect and respond to sound underwater, suggesting that some modifications of the ear may assist foraging or other behaviors below the surface. In air, the tympanic middle ear acts as an impedance matcher that increases sound pressure and decreases sound vibration velocity between the outside air and the inner ear. Underwater, the impedance-matching task is reversed and the ear is exposed to high hydrostatic pressures. Using micro- and nano-CT (computerized tomography) scans of bird ears in 127 species across 26 taxonomic orders, we measured a suite of morphological traits of importance to aerial and aquatic hearing to test predictions relating to impedance-matching in birds with distinct aquatic lifestyles, while accounting for allometry and phylogeny. Birds that engage in underwater pursuit and deep diving showed the greatest differences in ear structure relative to terrestrial species. In these heavily modified ears, the size of the input areas of both the tympanic membrane and the columella footplate of the middle ear were reduced. Underwater pursuit and diving birds also typically had a shorter extrastapedius, a reduced cranial air volume and connectivity and several modifications in line with reversals of low-to-high impedance-matching. The results confirm adaptations of the middle ear to aquatic lifestyles in multiple independent bird lineages, likely facilitating hearing underwater and baroprotection, while potentially constraining the sensitivity of aerial hearing.

## Introduction

Hearing is an important sensory modality of many animals, facilitating communication, navigation and detection of predators and prey. The tympanic middle ear of terrestrial vertebrates improves the transfer of sound energy from the outside of the ear to the inner ear, functioning as an ‘impedance matcher’^1–3^. Without the tympanic middle ear, most of the airborne sound would reflect at the air-body interface due to the higher impedance of body tissues relative to that of air. The form, shape and structure of the middle ear, however, is highly variable, reflecting the evolutionary history of vertebrates, including diversity of selection pressures and other contingencies (e.g. mechanical constraints, developmental or phylogenetic histories)^3^. For example, amphibious animals may face the challenge of satisfying hearing requirements in physically distinct terrestrial and aquatic environments, but the extent to which their auditory anatomy reflects their diverse lifestyles remains unclear.

For aquatic animals, augmenting hearing sensitivity underwater depends on increasing the vibration velocity of the sound, rather than the pressure^4–6^. This is because the acoustic impedance of water is slightly higher than that of the inner ear^4,5^. For aerial hearing, the tympanic middle ear minimizes the impedance mismatch through a larger area of the tympanic membrane relative to the footplate of the middle ear bone area, which increases the pressure (quantified as the ‘area ratio’), and also, through lever mechanisms that simultaneously increase the pressure and decrease the vibration velocity (quantified as ‘lever ratios’)^1–3,7^. A terrestrial tympanic middle ear might therefore be sub-optimally designed for underwater hearing because it would tend to increase the impedance mismatch between the water and inner ear. Likewise, an aquatic ear specialized for amplifying vibrations would lack the low-to-high impedance matching needed for detecting airborne sound^6^. Vertebrates with amphibious lifestyles, such as anurans, pinnipeds, crocodilians, most turtles, and many birds may therefore face a challenge in maintaining aerial hearing abilities if the ear is simultaneously adapted for hearing underwater^8–10^. Furthermore, diving species require modifications to protect delicate ear structures from hydrostatic pressure^11^. Modifications to the ear for performing in aquatic environments could therefore constrain aerial hearing sensitivity, and anatomical data on the ears of some amphibious taxa support such a constraint. Specifically, low area ratios or lever ratios have been described for turtles, pinnipeds, aquatic frogs and some aquatic birds^4,6,12^.

Aquatic birds are ideal models for examining evolutionary changes in a terrestrial auditory system upon return to aquatic lifestyles. While most diving and wading birds form a common clade, the Aequorlitornithes, transitions between terrestrial and aquatic lifestyles have occurred in several orders within this clade, and include both diving and non-diving members (e.g., Procellariiformes, Pelecaniformes, Charadriiformes). Aquatic foraging also evolved separately in diving ducks within the Anseriformes^13^. Among aquatic birds, many species forage just below the surface for brief periods whereas others plunge into the water at high speeds^14^, exposing the ear to high impact forces and rapid changes in ambient pressure between the outer ear and air-filled middle ear, presenting a risk for ear barotrauma. Some species can dive more than 100 m^15^, exposing the ear to an additional 10 atm of hydrostatic pressure. At increasing depths, visual cues become limited due to low light [e.g.,^16^], and sound can provide a more reliable sensory signal for detecting and pursuing prey or listening for environmental feedback^17,18^. Indeed, several studies support the use of hearing underwater in diving birds. These include the response to underwater vocalizations of predators and seismic noise in the African penguin *Spheniscus demersus*^19,20^, the production of vocalizations underwater in several penguin species^21^, and the detection of underwater sound in the gentoo penguin *Pygoscelis papua*, long-tailed duck *Clangula hyemalis*, great cormorant *Phalacrocorax carbo* and common murre *Uria aalge*^22–24^. Nonetheless, maintaining the ability to hear above water and on land is essential for aquatic birds as most species use acoustic signals for communication and reproduction^25,26^. Aerial hearing sensitivities of aquatic birds are, however, varied, suggesting a potential link between an aquatic lifestyle and reduced aerial hearing ability in some species^27,28^. The sensitivity of underwater hearing has only been measured in detail for the great cormorant, which has underwater hearing thresholds that are as low, or even lower, than the thresholds measured in air^17,28,29^. Furthermore, these studies reported several anatomical specializations in the middle ear likely involved in underwater hearing^16^.

**Figure 1:**
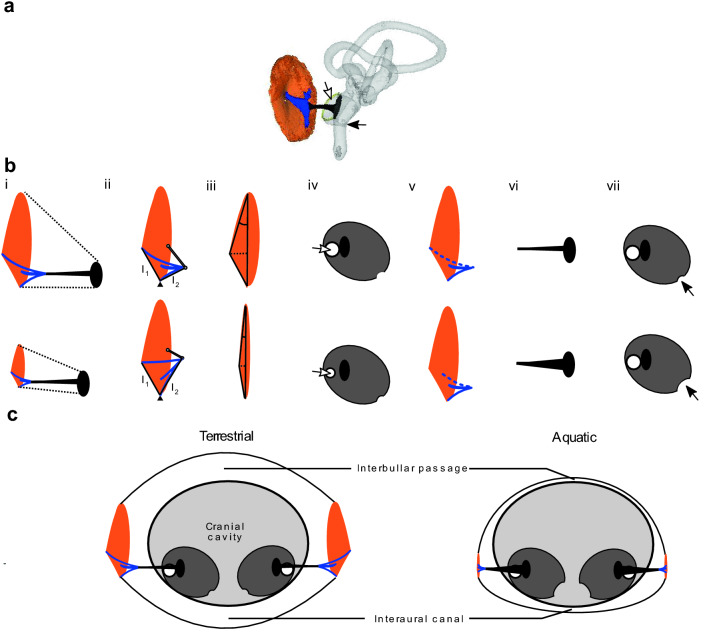
(**a**) Three-dimensional rendering of a typical terrestrial bird ear built from a micro-CT scan of a rock dove *Columba livia*, highlighting the tympanic membrane (orange), the trifurcated and cartilaginous extracolumella (blue), the columella bone (black), the inner ear (grey), the round window (white arrow) and cochlear aqueduct (black arrow). (**b**) Hypothesized anatomical differences between a bird ear typical of terrestrial species and thus adapted for aerial hearing (top row) versus one adapted for underwater hearing (bottom row). From aerial to aquatic, we expect (i) a reduction of the tympanic membrane-to-columella footplate area ratio (shown as a reduction in tympanic membrane area), (ii) a reduced offset (thin grey line) of the columella from the center of the eardrum, resulting in a smaller lever ratio (l_1_ and l_2_ indicate the lever arms), (iii) a flattened tympanic membrane, quantified as the height of the umbo and the angles at the periphery relative to the base plane of the tympanic membrane, (iv) a reduced area of the round window to raise total inner ear impedance, (v) a shorter extrastapedius, here used as a proxy of overall extracolumella stiffness (dotted line), (vi) hypertrophication of the columella, and (vii) enlargement of the cochlear aqueduct (represented by a curved indentation). (**c**) Summary of hypothesized differences in the ear of terrestrial (left) vs aquatic birds (right), combined with expectation of reduced cranial air and connectivity, interaural canal and interbullar passage between the two ears.

The bird middle ear is a single-ossicle structure, the columella auris, with a cartilage extracolumella connecting the tympanic membrane to the columella. The tympanic membrane has a convex point (the umbo), that arises due to the central process of the extracolumella (the extrastapedius)^30^. We hypothesize that aquatic birds may show a reversal of the impedance matching function of the middle ear by promoting increased vibration velocity via changes in anatomical structures (described in Fig. 1). For example, a smaller tympanic membrane-to-columella footplate area ratio would reduce the pressure augmentation (Fig. 1b-i), and two types of lever action could also be modified. The first lever involves a fulcrum at the edge of the tympanic membrane, where the ratio of the lever arms (one arm from fulcrum to columella tip and the other from fulcrum to extracolumella tip) is directly proportional to the vibration velocity (a second-order lever)^1^. A more centrally placed tip of the columella in aquatic species would reduce the lever action by reducing the ratio of lever arm lengths (Fig. 1b-ii)^2^. Modification to the second lever may involve flattening the convex point of the tympanic membrane, reducing the ‘catenary lever’ effect produced by the conical eardrum shape, and increasing vibration velocity^30,31^ (Fig. 1b-iii). A final way to potentially reduce the impedance mismatch underwater is through a reduced round window area (Fig. 1b-iv), making the ear impedance closer to that of the water; but, such an effect would also likely reduce aerial sensitivity. Some of these reversals, such as a lower tympanic membrane-to-columella footplate area ratio^32–34^ (electronic supplementary material S1) and flatter eardrums^28,35^, have been described anecdotally in aquatic birds, but have never been quantified and compared in a systematic way nor with appropriate allometric and phylogenetic contextualization.

Adaptation to aquatic life in birds might also affect the mass and stiffness of the middle ear (Fig. 1b-vi), which can influence frequency-dependent vibration transmission and thus aerial hearing sensitivity^7^. In seals and cetaceans, the hypertrophication and mineralization of the middle ear ossicles are considered underwater hearing adaptations^36,37^. Thickening of the extracolumella is found in turtles and *Xenopus* frogs, which have cartilage ‘tympanic discs’ rather than thin tympanic membranes^6,38^. In the great cormorant, the extracolumella is plate-like in contrast to its usual trifurcated shape^28^, making the ear stiffer, increasing footplate vibration relative to eardrum vibration, a likely advantage for underwater hearing^6^. A stiffer extracolumella may also be associated with lower pressure gain^30^.

Cranial air volume may also change with increasing aquatic adaptation, which can affect ear stiffness or the degree of interaural connectivity (Fig. 1c). In a typical terrestrial bird ear, the middle ear cavity is contiguous with a network of air-filled paratympanic sinuses that invaginate the cranium and connect the ears through one or more interaural connections^39^. These connections enable cross-talk between the ears and enhance directional hearing^39^. The post-cranial skeleton in diving birds typically shows reduced pneumaticity relative to terrestrial birds^40^, which may result in smaller overall cranial air volumes and reduced interaural connection. Among all birds studied to date, the interaural connection is known to be absent only in penguins (Spheniscidae)^41^.

Comparisons of morphological data that define these structure–function relationships in an allometric and phylogenetic context are essential to discern potential adaptations to underwater hearing in birds that have adopted amphibious lifestyles. In this study, we use micro- and nano-CT (computerized tomography) scans of the middle ears of 127 bird species to test whether a suite of morphological structures expected to affect underwater and aerial hearing capability differ in aquatic compared to terrestrial species. Specifically, we focus on traits related to reversal of low-to-high impedance-matching, the mass and stiffness of the middle ear, interaural connectivity, and inner ear openings (Fig. 1). Some modifications of the ear may relate to both underwater hearing and protecting the ear (e.g., increased pressure at depth or during plunge dives), and therefore we tested whether variation in specific ear structures of aquatic birds relates to diving capabilities and aquatic foraging mode (i.e., shallow surface-foraging vs. plunge-diving vs. underwater-pursuit). Commonalities in ear morphology in separate aquatic lineages that are distinct from those of terrestrial species would suggest convergent evolution in response to adoption of an aquatic lifestyle, and strengthen our functional interpretations.

## Methods

### Sampling of specimens

**Figure 2:**
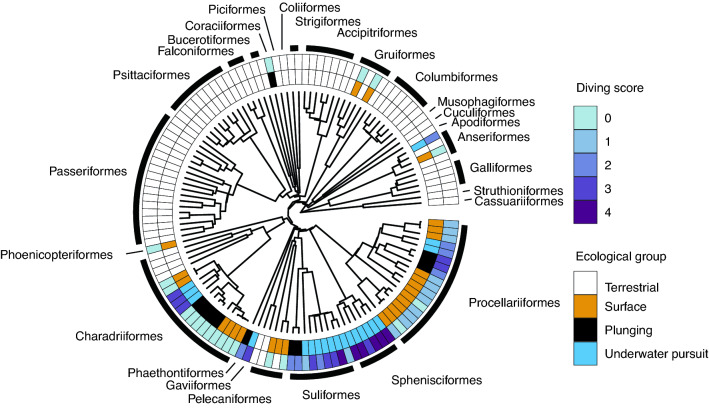
Phylogeny of species included in this study, labelled by four ecological groupings (terrestrial, surface-foraging, plunge-diving, and underwater pursuit) (inner ring), and classification of dive capability with highest point scores corresponding to deepest divers (outer ring). The phylogeny was constructed using a backbone based on published trees^13^ and species-level relationships using a ‘Hackett Stage 2’ phylogeny available online (www.birdtree.org).

Heads of naturally deceased adult birds were collected post-mortem with the exception of 5 specimens taken from an online source (Morphosource.org; full details in electronic supplementary material S2). Our study included micro- and nano-CT scans of heads from 135 birds representing 127 species (69 terrestrial and 58 aquatic species) and 26 taxonomic orders (Fig. 2). For most species, we only obtained a single specimen, but ear morphology is typically conserved within species^42^. When multiple specimens were available for a given species, the mean value was used in further analyses. Head mass was measured after decapitation, performed as close as possible to the base of the skull.

### Micro- and nano-CT scanning

Heads were stored frozen at − 20 or − 40 °C and then thawed overnight in a refrigerator before scanning. We used a micro-CT scanner (Phoenix v|tome|x L 240, General Electric, Wunstorf, Germany) and nano-CT scanner (Nanotom S, General Electric) at the Central Analytical Facility, Stellenbosch University^43^, and a micro-CT scanner (XTH225 ST, Nikon, Tokyo, Japan) at the Cambridge Biotomography Centre, University of Cambridge. Scans were restricted to the caudal region of the head to achieve the highest possible resolution of the ear relative to head size, while also simultaneously visualising the cranial airspace. Specimens completed a full 360-degree scan rotation at mean voltage of 100 kV (range: 60–170 kV), mean current of 209 µA (range: 100–300 µA), achieving a final mean voxel resolution of 28 µm (range: 6.0–110 µm). Full details of scan parameters are provided in electronic supplementary material S2.

### Morphological measurements

Full details of measurement methods are given in electronic supplementary material S3. Morphological measurements were taken from the generated 16-bit volumes using dedicated graphics software (Volume Graphics VGSTUDIO MAX version 3.2 software and 3D Slicer version 4.10.1, www.slicer.org^44^). We took 14 measures from each of the scans. Seven measures related to impedance mismatch, four of which related to input areas (tympanic membrane, columella footplate, the ratio between the tympanic membrane and columella footplate, and the round window). The remaining three related to lever action, including the umbo height, the tympanic membrane angle to quantify conical protrusion relating to the catenary lever, and the distance of columella offset from the center of the tympanic membrane quantifying the second-order lever.

The remaining measures involved quantification of the extracolumella (extrastapedius length), columella (length and volume), cranial air volume and connectivity, and the area of the cochlear aqueduct, an opening between the bony labyrinth and cranial cavity. The extracolumella of birds is flexible, and its central process, the extrastapedius (Fig. 1), undergoes significant bending^30,45^. Extrastapedius length was therefore expected to be of importance to the total stiffness of the middle ear and the length and volume of the columella of significance for its mass and inertia. Cranial air volume and two measurements of interaural connectivity were measured at the back of the head. The connectivity of the air passages through the inferior portion of the braincase (interaural canal, hereafter IAC), and superior to the foramen magnum (interbullar passage, hereafter IBP), were each scored as an ordered factor with three levels: ‘low’ signifies complete air-filled opening, ‘medium’ signifies opening in the bone but connection filled with soft tissues, and ‘high’ indicates lack of any pneumatic opening in the bone. Finally, cochlear aqueduct area was measured, motivated by larger cochlear aqueducts found in several aquatic birds^46^. Cranial air volume, columella volume, and columella length were measured directly from VGStudio and 3D Slicer software, whereas other lengths, angles, and areas were measured in R version 4.0.2^47^ from three-dimensional coordinates taken from 3D Slicer, with custom codes (see data, code and materials section and electronic supplementary material S4). Mean repeatability of nine different morphological variables ranged from 0.61 to 0.99 (intra-class correlation coefficient) using six individuals of Salvin’s prion *Pachyptila salvini* and seven individuals of rock dove *Columba livia* (see details in electronic supplementary material S5).

### Ecological classifications

We assigned species to four ecological groups based on the species predominant foraging strategy: terrestrial, surface foraging, plunge diving, or underwater pursuit (Fig. 2; electronic supplementary material S6). Terrestrial species have no regular association with water in terms of foraging behaviour. Surface-foraging species forage at the water surface or make very shallow dives, encompassing dipping, pattering, and surface-seizing feeding modes^48^. Plunge-diving species included both ‘pursuit-plunging’ species (e.g., gannets, shearwaters) and ‘surface-plunging’ species (e.g., terns). The underwater-pursuit category encompassed pursuit-diving and surface-swimming feeding modes (e.g., penguins, cormorants). Ears of surface-foraging groups were expected to show the least modification related to aquatic lifestyle. Plunge-diving species were included as a separate group to determine whether plunging correlates with distinct ear structures. Underwater-pursuit species were expected to exhibit the most pronounced ear modifications associated with enhanced underwater hearing.

We further classified aquatic birds according to diving capability to explore whether dive depth, and the associated need to protect the ear from high hydrostatic pressure, predicts specific ear structures. Dive-depth metrics were compiled from the literature for as many species as possible (electronic supplementary material S6) and ranked according to a 0 to 4 point score of overall maximum diving depth: 0 for non-diving species (< 1 m), 1 for shallow divers (1–10 m), 2 for intermediate divers (10.1–30 m), 3 for deep divers (30.1–100 m), and 4 for very deep divers (> 100 m). We used a dive score rather than using the variable maximum depth to avoid possible biases due to small sample size in a few studies or measurement error at shallow depths (see notes in electronic supplementary material S6).

### Statistical analyses

All statistical analyses were conducted in R version 4.0.2^47^ and incorporated a recent phylogeny of birds^13^ to resolve the deep relationships among bird orders (supplemental material of^49^), combined with the species-level phylogeny based on a ‘Hackett Stage 2’ phylogeny available online (www.birdtree.org). In some cases (*n* = 14), the species had to be substituted by the closest relative to build the phylogeny (electronic supplementary material table S7).

We performed a phylogenetic principal components analyses (pPCA), using the *phytools* package v. 0.7-70^50^, to ordinate all of the morphological measures and assess associations between measurements and groups based on ecology, diving capability and taxonomic order in a multivariate anatomical space. To correct for effects of head size, the input values for the pPCA were the residual values of phylogenetic generalized least squares (PGLS) regressions between each log-transformed ear measure and log-transformed head mass^51^.

We then used separate PGLS regressions to test whether auditory traits related to ecological groups, while controlling for head mass as a covariate and accounting for the non-independence of data due to phylogenetic relationships among species. Each PGLS regression estimated a maximum likelihood value of phylogenetic signal (Pagel’s λ) using the model residual errors. If there is low phylogenetic signal in the residuals, the model is equivalent to an ordinary least squares regression, whereas if it is close to 1, the variance–covariance matrix is related to the structure of the phylogeny. All continuous variables were log-transformed^52^ prior to PGLS regression analyses conducted using the *caper* package v. 1.0.1^53^. PGLS models were checked for normality and homoscedasticity of residuals.

For each auditory trait, three PGLS models were run: head mass alone as the main predictor, head mass plus the ecological group variable, and an interaction between the two. Terrestrial species were the reference group in these analyses. A second set of analyses focused on differences among aquatic birds only (surface-foraging, plunge-diving, and underwater-pursuit) and diving capability. For these analyses, the same three PGLS models were run for each auditory measure, except that surface-foraging was used as the reference group. In addition, two extra models including dive score were added to the selection process, namely head mass + dive score and head mass × dive score. For each set of analyses, we used Akaike information criterion adjusted for small sample sizes (AICc) to select the best-supported model among those tested for each ear measure. The best model was defined as the most parsimonious model (i.e., with fewest estimated parameters) within ΔAICc < 2 from the model with the lowest AICc score. If ecological group was in the best-supported model, significant differences between each ecological group and the reference group were tested using the ANOVA table output and an alpha level of 0.05.

The association between aquatic foraging mode and a reduction in interaural connection was tested using a Bayesian phylogenetic ordinal regression using a logit link function^54^, and controlling for phylogenetic relatedness. This regression analysis was implemented using the *brms* package v 2.14.4^55^ in R with default priors and 6000 iterations on two Monte Carlo chains. The null hypothesis of no difference in likelihood of the closure of the interaural canal (IAC) or interbullar passage (IBP) was rejected if the 95 % credibility interval did not include zero.

### Ethics approval

Specimen collection complied with the CapeNature permit CN-44-59-5360, and TOPS permit # S 29852, and the South African DEA marine research permit # RES2020/81. Specimen collection at South Georgia was carried out with permission of the Government of South Georgia and the South Sandwich Islands, and collection from Marion Island with permission from South African National Antarctic Programme (#110738 and #093071).

## Results

### Phylogenetic principal components analyses

**Figure 3:**
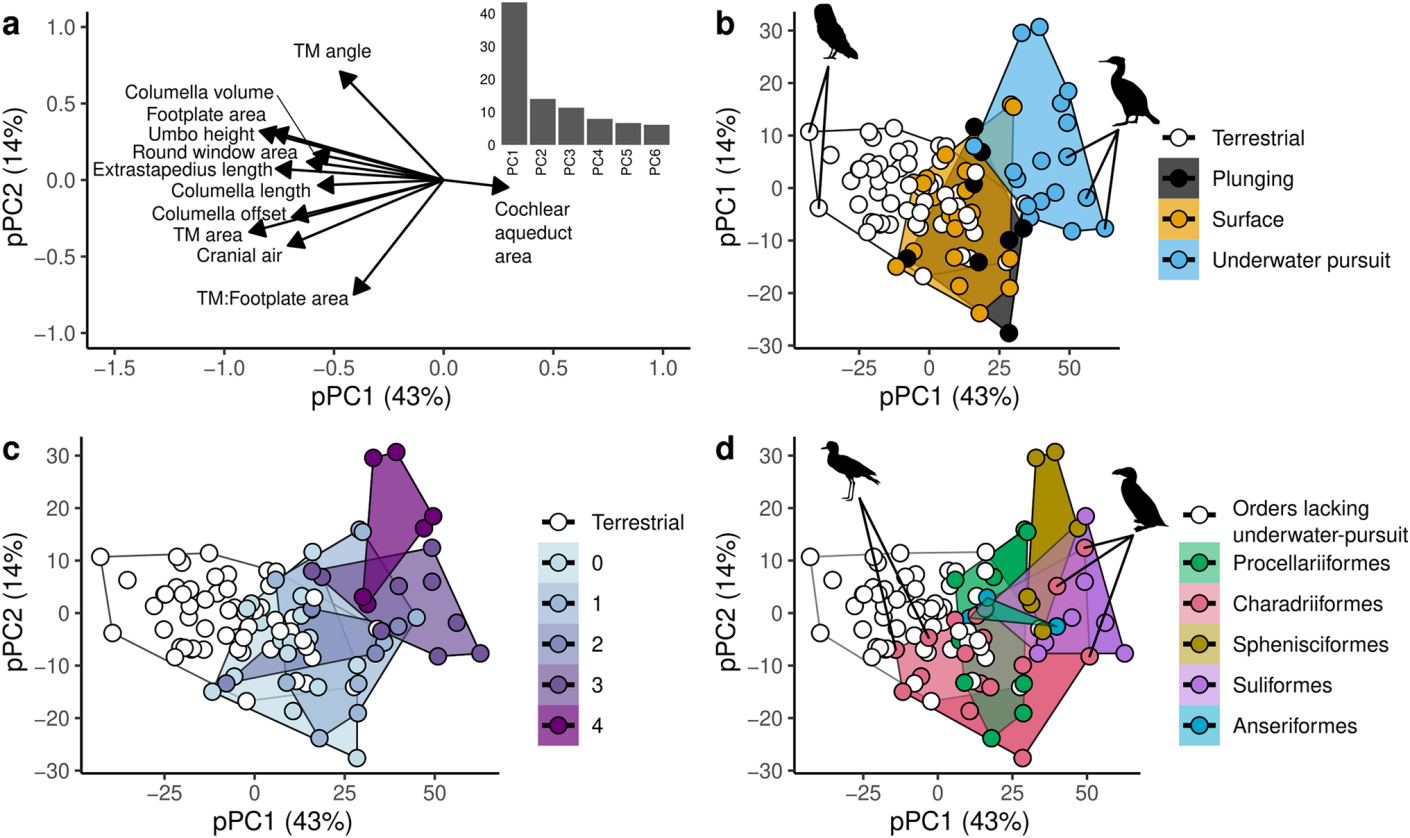
(**a**) Loading plots for pPC1 and pPC2 (TM stands for tympanic membrane) with inset bar plot of percent variance explained for first six principal components. (**b**) Scatterplot of pPC1 and pPC2 grouped by ecological groups. Several extreme scores are highlighted: owls (*Bubo africanus* and *Tyto alba*) on the negative pPC1 end and *Phalacrocorax* cormorants (*P. lucidus*, *P. capensis*, *P. neglectus*) on the positive pPC1 end. (**c**) Scatterplot of pPC1 and pPC2 grouped by dive score with increasing value of pPC1 indicating smaller values of most morphological measurements for a given head mass. (**d**) Scatterplot of pPC1 and pPC2 grouped by orders with at least one underwater-pursuit species. The diversity within Charadriiformes is highlighted by low pPC1 scores in two terrestrial species (*Burhinus capensis*, *Vanellus coronatus*) and high pPC1 scores in three diving species (*Alca torda*, *Fratercula arctica*, *Cepphus grylle*). Silhouette images provided by PhyloPic’s database (http://phylopic.org/), see electronic supplemental materials S8 for detailed credits.

The top two principal components, pPC1 and pPC2, explained 43 % and 14 % of the variance, respectively. Most of the auditory metrics were correlated with each other and related to low pPC1 loadings (Fig. 3a). Regarding pPC2, the tympanic membrane-to-columella footplate area ratio was associated with negative loading values while a measure of conical protrusion, the mean tympanic membrane angle, was associated with positive loading values.

All three aquatic groups scored higher on pPC1 than did the terrestrial species (Fig. 3b). Underwater-pursuit species showed greatest divergence from terrestrial species along pPC1, whereas surface-foraging and plunge-diving had intermediate values between terrestrial and underwater-pursuit species. Grouping by dive capability revealed that greater dive score was associated with higher pPC1 and pPC2 values (Fig. 3c). When grouped by orders that include at least one underwater-pursuit species, Suliformes and Sphenisciformes scored high on pPC1 (Fig. 3d). Charadriiformes, an ecologically diverse group that includes both terrestrial and underwater-pursuit members, showed the greatest spread of pPC1 scores for any order, in line with aquatic versus terrestrial ecologies within that group. Anseriformes and Procellariiformes showed intermediate values of pPC1 scores with a relatively greater degree of overlap with terrestrial species than both Suliformes and Sphenisciformes.

### Phylogenetic regression analyses

Full details of statistics for PGLS analyses are available in electronic supplementary material S9. When aquatic versus terrestrial species were compared, the best-supported models for most morphological measurements included head mass and ecological group as main effects, with no interaction terms (Table 1). There were a few exceptions to this pattern. Head mass alone was the best single predictor of cochlear aqueduct area and columella volume, while models that included an interaction between head mass and ecological group best explained columella length and tympanic membrane-to-footplate area ratio. When ecological group was significant (83 % of traits measured), underwater-pursuit species differed significantly from terrestrial species except for columella length, whereas surface-foraging and plunge-diving were each distinct from terrestrial species for only a unique subset of measurements (Table S9.2 and see electronic supplementary material S10 for examples of species differences in micro-CT slices and 3D renderings).

**Table 1:** Best supported PGLS models from AIC analysis, with adjusted R^2^ and Pagel’s λ, a measure of phylogenetic signal.

Species included in analysis	Anatomical category	Dependent variable	Model	Adjusted R^2^	λ
All species	Impedance-matching	log(tympanic membrane area)	log(head mass) + ecology	0.7	0.56
		log(footplate area)	log(head mass) + ecology	0.67	0.75
		log(tympanic membrane area/footplate area)	log(head mass) × ecology	0.27	0.16
		log(columella offset)	log(head mass) + ecology	0.53	0.4
		log(umbo height)	log(head mass) + ecology	0.34	0.43
		log(tympanic membrane angle)	log(head mass) + ecology	0.27	0.011
		log(extrastapedius length)	log(head mass) + ecology	0.59	0.65
		log(round window area)	log(head mass) + ecology	0.67	0.61
	Cochlear aqueduct	log(cochlear aqueduct area)	log(head mass)	0.31	0.85
	Air volume	log(cranial air volume)	log(head mass) + ecology	0.82	0.011
	Columella	log(columella length)	log(head mass) × ecology	0.85	0.65
		log(columella volume)	log(head mass)	0.78	0.46
Aquatic only	Impedance-matching	log(tympanic membrane area)	log(head mass) + dive score	0.78	0.0011
		log(footplate area)	log(head mass) + dive score	0.79	0.7
		log(tympanic membrane area/footplate area)	log(head mass) + dive score	0.22	0.045
		log(columella offset)	log(head mass) + dive score	0.4	0.63
		log(umbo height)	log(head mass) + dive score	0.13	0.0011
		log(tympanic membrane angle)	log(head mass)	0.024	0.0011
		log(extrastap-edius length)	log(head mass) + aquatic foraging lifestyle	0.5	0.75
		log(round window area)	log(head mass)	0.62	0.51
	Cochlear aqueduct	log(cochlear aqueduct area)	log(head mass) + dive score	0.55	0.92
	Air volume	log(cranial air volume)	log(head mass) + aquatic foraging lifestyle	0.78	0.2
	Columella	log(columella length)	log(head mass) × aquatic foraging lifestyle	0.87	0.8
		log(columella volume)	log(head mass) + aquatic foraging lifestyle	0.85	0.0011

**Figure 4:**
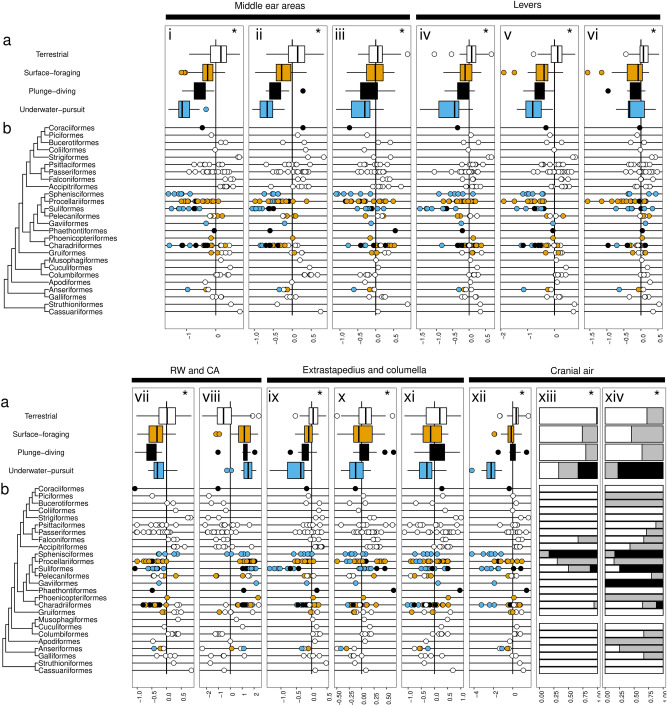
Relative sizes of auditory structures, adjusted for head mass and phylogenetic relatedness and grouped by ecological group (**a**) and order (**b**). Data are residuals of univariate PGLS regressions relating head mass with each respective ear measure, both as log-transformed variables. Negative values (left of the black vertical lines) indicate smaller values than expected for the bird’s head mass, and positive values indicating greater values than expected. Box plots show the distributions of residuals by ecological group in (**a**) and individual data points represent different species in (**b**). Colors indicate ecological groups (white for terrestrial, orange for surface-foraging, black for plunge-diving, blue for underwater-pursuit). (i) tympanic membrane area, (ii) columella footplate area, (iii) tympanic membrane-to-columella footplate area ratio, (iv) columella offset from center of tympanic membrane, (v) umbo height, (vi) tympanic membrane angle, (vii) round window area (RW), (viii) cochlear aqueduct area (CA), (ix) extrastapedius length, (x) columella length, (xi) columella volume (xii) cranial air volume. An asterisk is present if the best-supported model for that ear measure included ecological group. In panels xiii and xiv, bar plots indicate the proportions for three levels of cranial air connectivity for the interaural canal and interbullar passage, respectively (white for air-filled connection, grey for connection present but filled with soft tissue, black signifies no pneumatic opening in the bone).

Relative to terrestrial species of similar head mass, all three aquatic foraging modes showed smaller tympanic membrane area and several traits associated with lower lever action (reduced columella offset, tympanic membrane angle, umbo height) and smaller round window area (Fig. 4a; see electronic supplementary material, Table S9.1–2). Underwater-pursuit species showed the smallest tympanic membrane area (ca. 30 % of that of the terrestrial species; estimate =  − 1.20, s.e. = 0.14, *p* < 0.01), least offset columella (49 %; estimate =  − 0.72, s.e. = 0.11, *p* < 0.01), and shortest umbo height (40 %; estimate =  − 0.92, s.e. = 0.14, *p* < 0.01). The tympanic membrane angle of surface-foraging species (16°) differed the most from terrestrial species (22°) (estimate =  − 0.33, s.e. = 0.074, *p* < 0.01). Plunge-diving species had the smallest round window area, being 57 % of that of terrestrial species of similar head mass (estimate =  − 0.56, s.e. = 0.14, *p* < 0.01) (Fig. 4a-vii). There were no overall differences in tympanic membrane-to-footplate area ratio among ecological groups, but in underwater-pursuit species, the relationship between this area ratio and head mass was negative, whereas the corresponding slope was positive in other groups. The ratio intercept in underwater-pursuit species was higher than in terrestrial species, but the model explained relatively little variation (R^2^ = 0.27).

Plunge-diving and underwater-pursuit species had a smaller columella footplate area and extrastapedius length than terrestrial species of similar head mass, with the greatest differences observed for underwater-pursuit species; each measure was 60 % of the estimated value in terrestrial species (footplate area estimate =  − 0.50, s.e. = 0.12, *p* < 0.01; extrastapedius length estimate =  − 0.5, s.e. = 0.09, *p* < 0.01). There were no significant differences between surface-foraging species and terrestrial species (columella footplate area, *p* = 0.064; extrastapedius length, *p* = 0.15). In surface-foraging and plunge-diving birds, the slopes of the relationships between columella length and head mass were steeper than in terrestrial species, and the intercept in surface-foraging species was significantly lower than terrestrial species (Table S9.2).

Cranial air volume was moderately reduced in surface-foraging species (53 % of that of terrestrial species, estimate =  − 0.63, s.e. = 0.18, *p* < 0.01), and substantially reduced in underwater-pursuit species (7 %, estimate =  − 2.7, s.e. = 0.20, *p* < 0.01), but not significantly different in plunge-diving species (*p* = 0.15). Relative to terrestrial species, only underwater-pursuit was associated with more closed IACs (estimate = 20.56, s.e. = 18.83, 95 % credible interval = 5.20–72.80) and IBPs (estimate = 10.56, s.e. = 3.55, 95 % credible interval: 5.92–20.10). Complete closure of the IBP was observed only in underwater-pursuit species: penguins, several cormorants, the African darter *Anhinga rufa*, the great northern diver *Gavia immer*, and the black guillemot *Cepphus grylle* (Fig. 4a,b-xiv). The complete absence of IAC was found only in penguins and in the South Georgia shag *Leucocarbo georgianus* (electronic supplementary material Fig. S7).

When only aquatic species were compared, increased dive depth was associated with smaller tympanic membrane areas, footplate areas, tympanic membrane-to-footplate area ratios, columella offsets, umbo heights, and larger cochlear aqueduct areas (electronic supplementary material, Table S9.3–4). Underwater-pursuit species had extrastapedius lengths and cranial air volumes that were 68 % (estimate =  − 0.39, s.e. = 0.11, *p* < 0.01) and 11 % (estimate =  − 2.20, s.e. = 0.28, *p* < 0.01) of the values of surface foraging species, respectively, but there were no differences with plunge-diving species (both *p* > 0.40). The slope of the relationship between columella length and head mass was shallower in underwater-pursuit species relative to surface-foraging species (electronic supplementary material Table S9.4). Increased closure of both the IAC and the IBP was associated with underwater-pursuit behaviour (IAC: estimate = 17.55, s.e. = 17.24, 95 % CI = 1.46–72.89; IBP: estimate = 8.24, s.e. = 3.0, 95% CI: 4.14–16.26) and increasing dive depth (IAC: estimate = 2.6, s.e. = 2.6, 95 % CI = 0.63–11.31; IBP: estimate = 2.95, s.e. = 1.98, 95 % CI = 0.91–8.88).

## Discussion

Our study confirms that the middle ears of aquatic birds differ from those of terrestrial birds across multiple traits, with the greatest differences occurring in species with underwater-pursuit behaviour and deep diving capabilities. The Alcidae (Charadriiformes) and diving ducks (Anseriformes) are distantly related to cormorants and penguins, supporting an independent convergent evolution of ear morphology related to an aquatic lifestyle. In addition, the divergence of auditory structures within the ecologically diverse clades of the Charadriiformes suggests that adaptations of the ear are dependent on the environment. In line with this, the pPC1 axis in our analyses of ear morphology, which separates terrestrial and aquatic species, also relates to known aerial auditory sensitivities. Owls, which have the greatest aerial hearing sensitivity of birds^56^, scored very low on pPC1, whereas cormorants scored at the other extreme and are known to have relatively poor hearing in air^57^. There is a difference of 44 dB between the great cormorant at its best aerial hearing sensitivity and that of the western barn owl *Tyto alba* (35 dB and − 9 dB of sound pressure level, respectively^57,58^). These findings support a clear structure–function relationship for the ear of birds. Using our empirical dataset of ear morphology obtained from the wide diversity of species and ecological groups assembled, and accounting for head mass and phylogenetic relatedness among species, our comparative study is consistent with distinct patterns of auditory function among species with different lifestyles and suggests shared adaptations to underwater hearing in amphibious birds.

In line with our hypotheses, we found reversals of the standard terrestrial low-to-high impedance matching related to area ratio and lever mechanisms. Eardrum-to-footplate area ratios were smallest in deep-diving birds, while reduced levers were observed generally in all aquatic birds. The area ratio results are consistent with previous findings of lower area ratios in aquatic birds, including penguins, the group with the lowest area ratios^32–34^. Reversing both the catenary and second-order levers, a given vibration velocity of the eardrum will theoretically be associated with a greater vibration velocity of the footplate, resulting in increased sensitivity to underwater sound^6^. Levers that increase particle vibration velocity are described as an underwater hearing adaptation in *Xenopus* frogs^6^ and toothed whales^4,8^.

The reduced size of both the tympanic membrane area and the columella footplate area in underwater-pursuit and deep-diving species was more pronounced than differences in area ratio. This was unexpected, since the area ratio is assumed to be a major component of the impedance match^3^, and consequently hearing sensitivity. The area ratio also did not load highly on pPC1, which seems to have a higher correlation to aerial hearing sensitivity than pPC2, considering the extreme opposite points for owls and *Phalacrocorax* cormorants. The reductions in tympanic membrane and footplate areas are consistent with a recent scaling study that shows a relatively small footplate (relative to skull width) in the double-crested cormorant *Phalacrocorax auritus*, and small tympanic membrane in the African penguin^59^. While not affecting the impedance matching, reduced tympanic membrane and footplate areas may still affect sensitivity. If all else is held constant (i.e., area ratio, levers, mass and stiffness), a smaller footplate should displace a smaller total volume of inner ear fluid, and smaller tympanic membrane and footplates may have less mobility than larger ones^60^.

Aquatic species also showed traits that likely stiffen the ear, including a shortened extrastapedius length and a reduced cranial air volume. The most extreme case of extrastapedius reduction was in Suliformes (Fig. 4b-ix), which supports the flattened, plate-like eardrums described for the great cormorant^28^, and relatively short extrastapedius that was recently noted in African penguins^59^. Shortened extrastapedius length may reflect a general trend for a more robust middle ear in diving birds, following patterns of the increasing extracolumella in turtles, *Xenopus* frogs, and great cormorants. Cranial air volumes were substantially reduced in underwater-pursuit birds relative to terrestrial birds. Similarly, in turtles, aquatic taxa have smaller cranial middle ear air cavity volumes^61^. Underwater-pursuit behaviour and increasing dive capability was associated with a lower degree of air-filled connectivity between the ears. In addition to penguins (see^41^), the South Georgia shag, a member of the exceptionally deep-diving *Leucocarbo* genus^62^, also lacks the interaural canal, supporting an association between deep diving and reduction of cranial pneumaticity. Nonetheless, some degree of interaural connectivity was found in diving birds, although to a lesser extent than in terrestrial birds. Narrowing the interaural connectivity should lower the frequency range over which the ears have enhanced interaural cues by internal coupling of the ears^63^. Reduced interaural connection, however, does not necessarily mean that directional hearing is impaired as birds could rely increasingly on neural mechanisms rather than the inherent directionality provided by internally coupled ears. Penguins have to localize sounds in noisy colonies on land and some connectivity may be maintained through the completely ossified eustachian tubes that open into the nasopharynx^41^.

Protection of the ear from barotrauma can confound adaptations associated with underwater hearing. First, birds that spend more time underwater or those that dive at depths where visual cues are impaired, are more likely to have adaptations for underwater hearing. In addition, dive duration is associated with dive depth in our dataset (electronic supplementary material S11). Second, a reduction in pressure amplification, and increase in stiffness, may be beneficial for both baroprotection and enhanced underwater hearing. Furthermore, the very small tympanic membrane area in the underwater-pursuit group might be a consequence of narrowing the ear canal to facilitate its collapse during dives. The high hydrostatic pressure closes the ear canal upon descent of the water column in cormorants and auks^28,33^. We also found that penguins had a narrowing of the ear canal (electronic supplementary material S12), which closes by contraction of specific muscles in the external ear^64^. This narrowing may place an upper limit on the tympanic membrane area. A smaller columella footplate in deep-diving birds, might therefore compensate for the smaller tympanic membrane to maintain the tympanic membrane-to-columella footplate area ratio required to offset the impedance-mismatch of aerial hearing.

The lack of hypertrophication of the columella total size or footplate area in underwater-pursuit birds suggests that birds have taken a different evolutionary trajectory relative to phocids in their adaptation to aquatic environments^5^. The need to maintain sensitivity to higher pitched vocalizations in air could constrain the benefit of using such a mechanism underwater, as a heavy ossicle limits high frequency hearing^37^, particularly when combined with small eardrum areas and low lever ratios. Our data confirm previous observations of large cochlear aqueducts in aquatic birds^46^, indicating a substantial ‘third window’ to the inner ear, which may be of significant relevance for underwater hearing^65^. Waterbirds had large aqueducts (represented by members of the clade encompassed by Sphenisciformes to Charadriiformes; Fig. 4b-viii), and among diving birds, a larger cochlear aqueduct was associated with deeper diving capability. Enlarged cochlear aqueduct openings may allow for better coupling of fluid vibrations between the cranial cavity and the inner ear, facilitating bone-conduction hearing^65^ or sound localization underwater^46^. Finally, there may be a limit in the degree to which reversals in the low-to-high impedance-matching traits augment aquatic hearing sensitivity. For example, the upper limits of velocity amplification of the middle ear via the lever mechanisms would involve flattening the eardrum and having the columella centrally positioned. Vibrations from air trapped in the middle ear, however, could set in motion a further increase in vibration velocity of middle ear structures underwater, a mechanism described in the ears of turtles and frogs^3,38^, and presumed to operate in aquatic birds too^28^.

We present the first systematic comparison of the middle ear, corrected for head mass and phylogeny, between aquatic and terrestrial birds. Our results suggest that aquatic lifestyles impose selective pressures on ear design, with most pronounced differences observed in deep-diving and underwater-pursuit species of bird. Distantly related underwater-pursuit species shared commonalities in ear morphology, supporting a convergent evolution across lineages. The reversal of low-to-high impedance-matching and changes in the extracolumella and cranial air cavity are consistent with findings in other amphibious animals, whereas the absence of a columella hypertrophication diverges from the pattern of middle ear ossification in phocid pinnipeds and some odobenids. We found several characteristics that are in line with amphibious hearing, supporting predictions based on the reversal of low-to-high impedance matching in aquatic birds (e.g., lever mechanisms), but remain cognizant that some structural changes may play a role for baroprotection (e.g., reduced tympanic membrane area associated with narrowing of the ear canal, large cochlear aqueduct), or for both baroprotection and underwater hearing (e.g., reduced cranial air volume). However, we recognize that changes could also result from relaxed selection on arial hearing due to decreasing time spent on land, without accompanying advantages to underwater hearing or baroprotection. Regardless of the ecological drivers of underwater ear modifications, a pressing line of enquiry will be to investigate if the characteristics uncovered in the highly aquatic species constraint their hearing sensitivity in air.

## Supplementary Information

Supplementary Information 1.

Supplementary Information 2.

Supplementary Information 3.

## Data Availability

Complete CT scans, with one representative per species, are available on Morphosource.org (https://www.morphosource.org/Detail/ProjectDetail/Show/project_id/1148). Morphosource specimen numbers and ark file IDs of all scans are given in electronic supplementary material S2. R code for morphological measurements are available at https://doi.org/10.5281/zenodo.4543752 and R code for statistical analyses are available at 10.5281/zenodo.4587146.

## References

[1] Saunders JC, Duncan RK, Doan DE, Werner YL, Dooling RJ, Fay RR, Popper AN (2000). The middle ear of reptiles and birds. Comparative Hearing: Birds and Reptiles.

[2] Manley GA (1995). The lessons of middle-ear function in non-mammals: Improving columellar prostheses. J. R. Soc. Med..

[3] Christensen-Dalsgaard J, Manley GA, Köppl C, Manley GA, Popper AN, Fay RR (2013). The malleable middle ear: An underappreciated player in the evolution of hearing in vertebrates. Insights from Comparative Hearing Research.

[4] Hemilä S, Nummela S, Reuter T (1999). A model of the odontocete middle ear. Hear. Res..

[5] Nummela S, Thewissen JGM, Bajpai S, Hussain T, Kumar K (2007). Sound transmission in archaic and modern whales: Anatomical adaptations for underwater hearing. Anat. Rec. Adv. Integr. Anat. Evol. Biol..

[6] Mason M, Wang M, Narins P (2009). Structure and function of the middle ear apparatus of the aquatic frog, *Xenopus laevis*. Proc. Inst. Acoust. Inst. Acoust. G. B..

[7] Mason MJ (2016). Structure and function of the mammalian middle ear. II: Inferring function from structure. J. Anat..

[8] Hemilä S, Nummela S, Reuter T (2010). Anatomy and physics of the exceptional sensitivity of dolphin hearing (Odontoceti: Cetacea). J. Comp. Physiol. A.

[9] Reichmuth C, Holt MM, Mulsow J, Sills JM, Southall BL (2013). Comparative assessment of amphibious hearing in pinnipeds. J. Comp. Physiol. A.

[10] Zeyl JN, Johnston CE (2015). Amphibious auditory evoked potentials in four North American Testudines genera spanning the aquatic–terrestrial spectrum. J. Comp. Physiol. A.

[11] Kooyman GL, Wursig B, Perrin WF (2009). Diving physiology. Encyclopedia of Marine Mammals.

[12] Smodlaka H, Khamas WA, Jungers H, Pan R, Al-Tikriti M, Borovac JA, Palmer L, Bukac M (2019). A novel understanding of phocidae hearing adaptations through a study of northern elephant seal (*Mirounga angustirostris*) Ear Anatomy and Histology. Anat. Rec..

[13] Prum RO, Berv JS, Dornburg A, Field DJ, Townsend JP, Lemmon EM, Lemmon AR (2015). A comprehensive phylogeny of birds (Aves) using targeted next-generation DNA sequencing. Nature.

[14] Chang B, Croson M, Straker L, Gart S, Dove C, Gerwin J, Jung S (2016). How seabirds plunge-dive without injuries. Proc. Natl. Acad. Sci..

[15] Pütz K, Wilson RP, Charrassin J-B, Raclot T, Lage J, Maho YL, Kierspel MAM, Culik BM, Adelung D (1998). Foraging strategy of king penguins (*Aptenodytes patagonicus*) during summer at the Crozet Islands. Ecology.

[16] White CR, Day N, Butler PJ, Martin GR (2007). Vision and foraging in cormorants: more like herons than hawks?. PLoS ONE.

[17] Hansen KA, Maxwell A, Siebert U, Larsen ON, Wahlberg M (2017). Great cormorants (*Phalacrocorax carbo*) can detect auditory cues while diving. Sci. Nat..

[18] Larsen ON, Radford C, Slabbekoorn H, Dooling RJ, Popper AN, Fay RR (2018). Acoustic conditions affecting sound communication in air and underwater. Effects of Anthropogenic Noise on Animals.

[19] Frost PGH, Shaughnessy PD, Semmelink A, Sketch M, Siegfried WR (1975). Response of jackass penguins to killer whale vocalizations. S. Afr. J. Sci..

[20] Pichegru L, Nyengera R, McInnes AM, Pistorius P (2017). Avoidance of seismic survey activities by penguins. Sci. Rep..

[21] Thiebault A, Charrier I, Aubin T, Green DB, Pistorius PA (2019). First evidence of underwater vocalisations in hunting penguins. PeerJ.

[22] Therrien SC (2014). In-air and underwater hearing of diving birds. DRUM.

[23] Hansen K, Hernandez A, Mooney TA, Rasmussen MH, Sørensen K, Wahlberg M (2020). The common murre (*Uria aalge*), an auk seabird, reacts to underwater sound. J. Acoust. Soc. Am..

[24] Sørensen K, Neumann C, Dähne M, Hansen KA, Wahlberg M (2020). Gentoo penguins (*Pygoscelis papua*) react to underwater sounds. R. Soc. Open Sci..

[25] Aubin T, Jouventin P (2002). Localisation of an acoustic signal in a noisy environment: The display call of the king penguin *Aptenodytes patagonicus*. J. Exp. Biol..

[26] Thiebault A, Pistorius P, Mullers R, Tremblay Y (2016). Seabird acoustic communication at sea: A new perspective using bio-logging devices. Sci. Rep..

[27] Crowell SE, Wells-Berlin AM, Carr CE, Olsen GH, Therrien RE, Yannuzzi SE, Ketten DR (2015). A comparison of auditory brainstem responses across diving bird species. J. Comp. Physiol. A.

[28] Larsen ON, Wahlberg M, Christensen-Dalsgaard J (2020). Amphibious hearing in a diving bird, the great cormorant (*Phalacrocorax carbo sinensis*). J. Exp. Biol..

[29] Johansen S, Larsen ON, Christensen-Dalsgaard J, Seidelin L, Huulvej T, Jensen K, Lunneryd S-G, Boström M, Wahlberg M, Popper AN, Hawkins A (2016). In-Air and underwater hearing in the great cormorant (*Phalacrocorax carbo sinensis*). The Effects of Noise on Aquatic Life II.

[30] Muyshondt PGG, Dirckx JJJ (2020). How flexibility and eardrum cone shape affect sound conduction in single-ossicle ears: A dynamic model study of the chicken middle ear. Biomech. Model. Mechanobiol..

[31] Tonndorf J, Khanna SM (1970). The role of the tympanic membrane in middle ear transmission. Ann. Otol. Rhinol. Laryngol..

[32] Frahnert S, Lindner M, Bendel E-M, Frahnert KH, Westphal N, Dähne M (2019). 3D-visualization of the ear morphology of penguins (Spheniscidae): Implications for hearing abilities in air and underwater. Proc. Meet. Acoust..

[33] Kartaschew N, Iljitschwe WD (1964). Über das Gehörorgan der Alkenvögel. J. Ornithol..

[34] Schwartzkopff J (1955). On the hearing of birds. Auk.

[35] Mills R (1994). Applied comparative anatomy of the avian middle ear. J. R. Soc. Med..

[36] Nummela S, Wägar T, Hemilä S, Reuter T (1999). Scaling of the cetacean middle ear. Hear. Res..

[37] Hemilä S, Nummela S, Berta A, Reuter T (2006). High-frequency hearing in phocid and otariid pinnipeds: An interpretation based on inertial and cochlear constraints. J. Acoust. Soc. Am..

[38] Christensen-Dalsgaard J, Brandt C, Willis KL, Christensen CB, Ketten D, Edds-Walton P, Fay RR, Madsen PT, Carr CE (2012). Specialization for underwater hearing by the tympanic middle ear of the turtle, *Trachemys scripta elegans*. Proc. R. Soc. B Biol. Sci..

[39] Larsen ON, Christensen-Dalsgaard J, Jensen KK (2016). Role of intracranial cavities in avian directional hearing. Biol. Cybern..

[40] Smith ND (2012). Body mass and foraging ecology predict evolutionary patterns of skeletal pneumaticity in the diverse “Waterbird” clade. Evolution.

[41] Ksepka DT, Balanoff AM, Walsh S, Revan A, Ho A (2012). Evolution of the brain and sensory organs in Sphenisciformes: New data from the stem penguin *Paraptenodytes antarcticus*. Zool. J. Linn. Soc..

[42] Cerio DG, Witmer LM (2019). Intraspecific variation and symmetry of the inner-ear labyrinth in a population of wild turkeys: Implications for paleontological reconstructions. PeerJ.

[43] du Plessis A, le Roux SG, Guelpa A (2016). The CT Scanner Facility at Stellenbosch University: An open access X-ray computed tomography laboratory. Nucl. Instrum. Methods Phys. Res. Sect. B Beam Interact. Mater. At..

[44] Fedorov A (2012). 3D Slicer as an image computing platform for the quantitative imaging network. Magn. Reson. Imaging.

[45] Claes R, Muyshondt PGG, Dirckx JJJ, Aerts P (2018). Deformation of avian middle ear structures under static pressure loads, and potential regulation mechanisms. Zoology.

[46] Kohllöffel LUE (1984). Notes on the comparative mechanics of hearing. II. On cochlear shunts in birds. Hear. Res..

[47] R Core Team. *R: A Language and Environment for Statistical Computing* (R Foundation for Statistical Computing, 2020). See https://www.R-project.org/.

[48] Ashmole NP, Farner DS, King JR (1971). Seabird ecology and the marine environment. Avian Biology.

[49] Cooney CR, Bright JA, Capp EJR, Chira AM, Hughes EC, Moody CJA, Nouri LO, Varley ZK, Thomas GH (2017). Mega-evolutionary dynamics of the adaptive radiation of birds. Nature.

[50] Revell LJ (2009). Size-correction and principal components for interspecific comparative studies. Evolution.

[51] Revell LJ (2012). phytools: An R package for phylogenetic comparative biology (and other things). Methods Ecol. Evol..

[52] Glazier DS (2013). Log-transformation is useful for examining proportional relationships in allometric scaling. J. Theor. Biol..

[53] Orme, D. *et al.**caper: Comparative Analyses of Phylogenetics and Evolution in R*. See https://CRAN.R-project.org/package=caper (2018).

[54] Bürkner P-C, Vuorre M (2019). Ordinal regression models in psychology: A tutorial. Adv. Methods Pract. Psychol. Sci..

[55] Bürkner P-C (2018). Advanced Bayesian multilevel modeling with the R package brms. R J..

[56] Dooling RJ, Lohr B, Dent ML, Dooling RJ, Fay RR, Popper AN (2000). Hearing in birds and reptiles. Comparative Hearing: Birds and Reptiles.

[57] Maxwell A, Hansen KA, Ortiz ST, Larsen ON, Siebert U, Wahlberg M (2017). In-air hearing of the great cormorant (*Phalacrocorax carbo*). Biol. Open.

[58] Dyson ML, Klump GM, Gauger B (1998). Absolute hearing thresholds and critical masking ratios in the European barn owl: A comparison with other owls. J. Comp. Physiol. A.

[59] Peacock J, Spellman GM, Greene NT, Tollin DJ (2020). Scaling of the avian middle ear. Hear. Res..

[60] Werner YL, Montgomery LG, Seifan M, Saunders JC (2008). Effects of age and size in the ears of gekkotan lizards: auditory sensitivity, its determinants, and new insights into tetrapod middle-ear function. Pflüg. Arch. Eur. J. Physiol..

[61] Foth C, Evers SW, Joyce WG, Volpato VS, Benson RBJ (2019). Comparative analysis of the shape and size of the middle ear cavity of turtles reveals no correlation with habitat ecology. J. Anat..

[62] Croxall JP, Naito Y, Kato A, Rothery P, Briggs DR (1991). Diving patterns and performance in the Antarctic blue-eyed shag *Phalacrocorax atriceps*. J. Zool..

[63] Christensen-Dalsgaard J (2011). Vertebrate pressure-gradient receivers. Hear. Res..

[64] Sadé J, Handrich Y, Bernheim J, Cohen D (2008). Pressure equilibration in the penguin middle ear. Acta Otolaryngol. (Stockh.).

[65] March D, Brown D, Gray R, Curthoys I, Wong C, Higgins DP (2016). Auditory anatomy of beaked whales and other odontocetes: Potential for cochlear stimulation via a “vibroacoustic duct mechanism”. Mar. Mammal Sci..

