# Supplementary material for: Aquatic birds have middle ears adapted to amphibious lifestyles
Source: Sci Rep. 2022 Mar 28;12:5251. doi: 10.1038/s41598-022-09090-3 (PMC8960762; doi:10.1038/s41598-022-09090-3)
Supplement: Supplementary file 1 — Supplementary Information 1. [file 41598_2022_9090_MOESM1_ESM.docx]

Contents

[S1 Area ratio values from the literature 1](#_Toc67649876)

[S2 micro-CT scan metadata (csv file) 2](#_Toc67649877)

[S3 Anatomical measurements 2](#_Toc67649878)

[S4 Anatomical calculations 6](#_Toc67649879)

[S5 Measurement repeatability 7](#_Toc67649880)

[S6 Details of ecological groupings (csv file) 7](#_Toc67649881)

[S7 Closest-related species used from birdtree.org phylogeny 8](#_Toc67649882)

[S8 Bird silhouette image credits 8](#_Toc67649883)

[S9 Tables of PGLS summary statistics for all models 9](#_Toc67649884)

[S10 Examples of anatomical differences in micro-CT scans 17](#_Toc67649885)

[S11 Dive depth and dive duration correlations 21](#_Toc67649886)

[S12 Narrow ear canal of king penguin 22](#_Toc67649887)

[References 22](#_Toc67649888)

# S1 Area ratio values from the literature


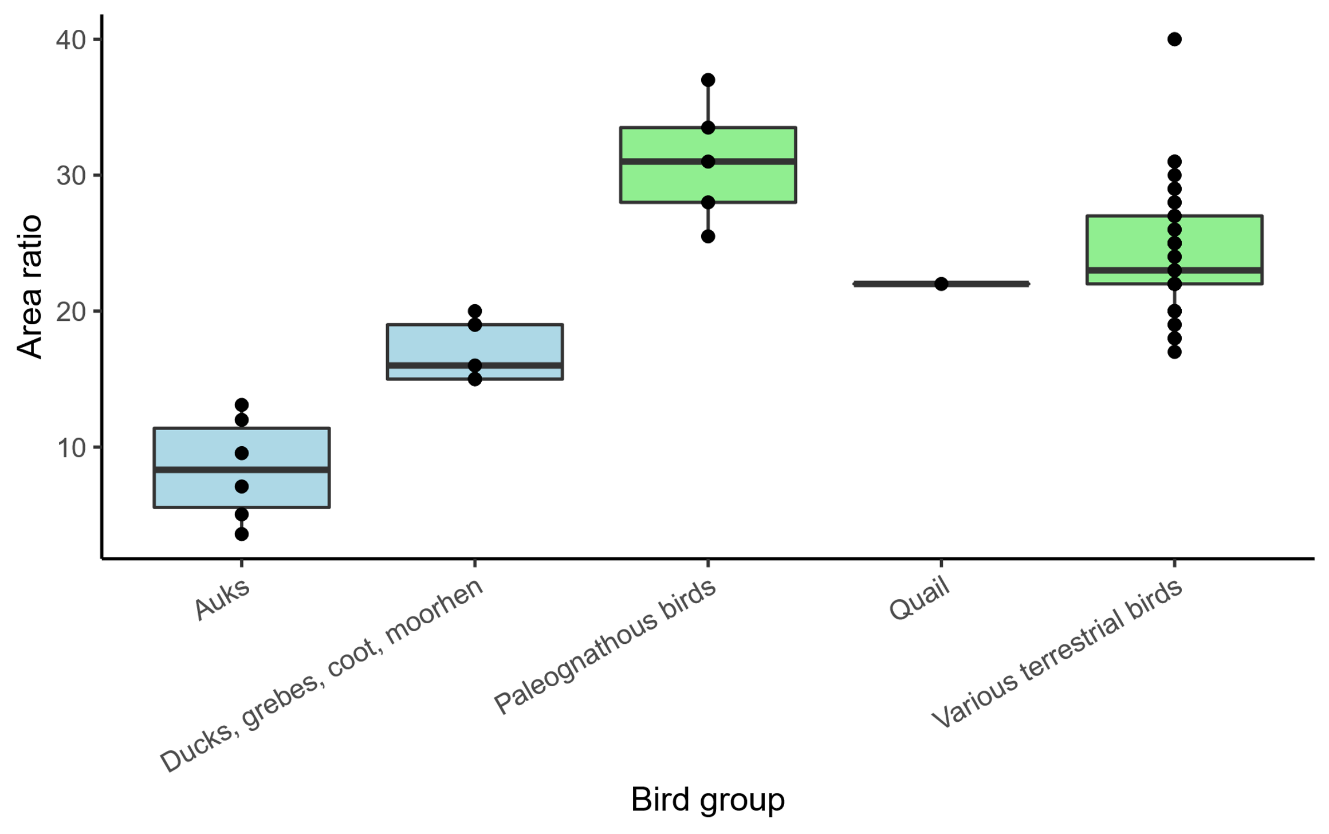


Fig. S1. Boxplots of area ratios (tympanic membrane-to-columella footplate), compiled from the literature, in aquatic (blue) and terrestrial (green) birds. Data are compiled from various terrestrial birds, ducks, coot, grebes, moorhen [1], auks [2], and paleognathous birds and quail [3]. Black dots are original data.

# S2 micro-CT scan metadata (csv file)

This file contains various scan parameters and details for each scan ID and its corresponding digital specimen on Morphosource. The file has the following columns: order, species scientific names, common name, specimen code, scan date, Morphosource specimen number, Morphosource file ark identifier, specimen type (either head, head in fluid fixative, or scan from digital repository), scan facility, scanner model, resolution (x), resolution (y), resolution (z), tube voltage (kV), tube current (uA), number of images acquired, acquisition time for each image, averaging (number of images taken at each step position during rotation, then averaged to reduce noise), filters used, rotation (in degrees), bit depth, and specimen source.

# S3 Anatomical measurements

The tympanic membrane area was computed using coordinates from the perimeter and the umbo (Fig. S2). To select the umbo landmark, the extrastapedius and tympanic membrane region was segmented and the umbo tip point was manually selected on the 3D model from the external surface of the tympanic membrane and using the extrastapedius as a guide. In most cases, the extrastapedius and tympanic membrane region could be automatically segmented using Isodata and Maximum Entropy algorithms available in 3D Slicer, but additional automatic algorithms or manual adjustment was required for 15 samples (12% of scans).

The perimeter of the tympanic membrane was selected where the membrane transitions to the soft tissue lining of the ear canal and the middle ear (Fig. S2). The tympanic membrane perimeter was traced with 20 to 50 3D points though the micro-CT slices, which were then converted to a smoothed curve containing 16 equidistant points using the digit.curves function of the *geomorph* package [4] in R. Total tympanic membrane area was then determined by summing the areas of the 16 triangles that connected each adjacent perimeter point with the umbo, with area computed using Heron’s formula for the area of the triangle (see section S4). The areas of the columella footplate, round window, and cochlear aqueduct followed a similar procedure, with slight modifications. Columella footplate perimeter was measured at its widest point from the 3D model of the columella in Meshlab software v. 2016.12 [5] using the ‘picked points’ tool, with the centroid of the 16 perimeter points used as the center point for the triangles. The boney perimeters of the round window and lateral opening of the cochlear aqueduct (i.e., as it exits from the bony labyrinth) were outlined from the micro-CT scans directly with centroid as a center point.


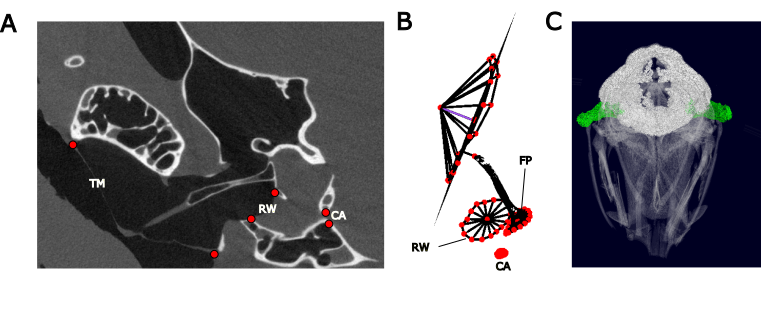


Fig. S2. (A) Example of a micro-CT slice through the ear showing the placement of perimeter points (red circles) for the cochlear aqueduct (CA), round window (RW), and tympanic membrane (TM). (B) 3D rendering of the ear points used for measurement calculations in red, and connecting black lines outlining the triangles used in area calculations (FP = columella footplate). Height of umbo is shown in purple in relation to the plane of best fit through the tympanic membrane perimeter points. (C) 3D rendering of internal air cavity volume (white) highlighting the interaural canal and interbullar passage. Ear canals shown in green.

The offset of the columella from the center of the eardrum was measured as the distance between its distal tip and the centroid computed from the 16 tympanic membrane perimeter points. The columella distal tip was selected from 3D models as the closest point on the columella relative to the umbo. The conical protrusion of the tympanic membrane was quantified two ways, by measuring the angles at the perimeter of the tympanic membrane and the height. First, the angles between the 16 lines connecting each perimeter point to the umbo and the base plane of the tympanic membrane was calculated, and the mean taken (calculations in electronic supplementary materials S4). Second, the height of the umbo was measured, defined as the distance from the umbo to the base plane fit to the tympanic membrane perimeter (Fig. S2).

Extrastapedius length was measured as the straight-line distance from the umbo (selected manually as defined above) to the distal tip of the columella. Columella length was measured as the straight-line distance from the most distal point of the columella (relative to the columella footplate) to the bottom of the columella footplate, where the columella shaft joins the footplate. Columella volume was determined from segmentations using the ‘maximum entropy’ automatic threshold algorithm in 3D Slicer. In 12 instances (9%), maximum entropy was unsuccessful and threshold was manually determined.

Cranial air volume was defined and measured as all the cranial pneumaticity in the back of the skull connected to the middle ear cavity, excluding the external auditory canal. First, the volume was oriented so the basicranium was horizontal, and then the region of interest was restricted to the head caudal to the joining of the carotid canals. Starting in the air region behind the eardrum, we used the ‘region growing’ tool in VGL studio software, set to ‘IsoData’ threshold algorithm, to segment air connected to the air cavity. Air in the external ear canal was excluded from this measurement by fitting the Gaussian plane of best fit through 10 to 15 points outlining the perimeter of the tympanic membrane and subtracting the ear canal from the total volume. Scans of 20 species were excluded from this analysis because the cranial air spaces were obstructed by fluid seepage. These also occasionally prevented the measurements from the tympanic membrane and extracolumella. The total sampling for each measure and for each species is shown in Fig. S3:


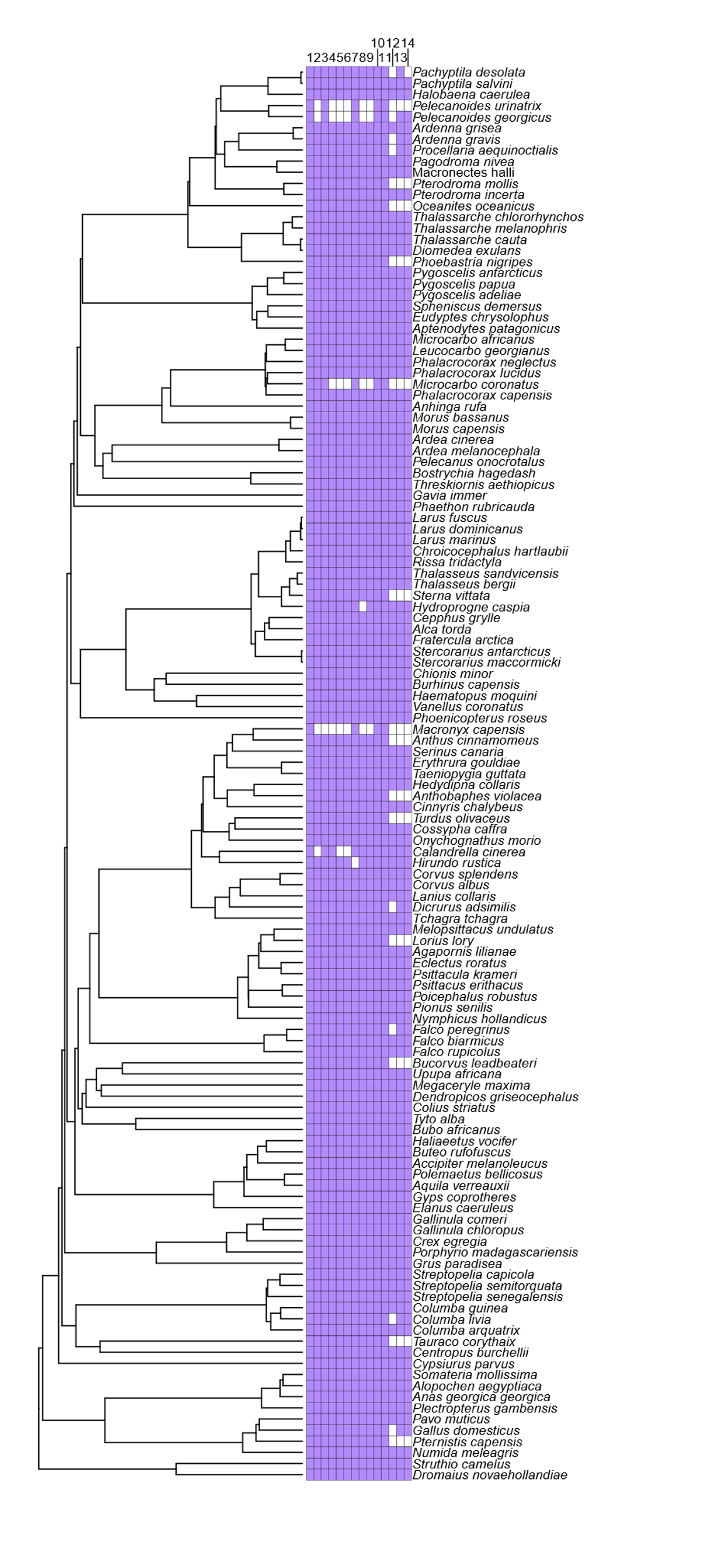


Fig. S3. The extent of scanning and data collection is shown in this heatmap next to the phylogeny, each column indicating a different ear measurement. White values indicate that data could not be measured due to poor specimen quality. 1: head mass, 2: tympanic membrane area, 3: footplate area, 4: columella offset, 5: mean tympanic membrane angle, 6: umbo height, 7: cochlear aqueduct area, 8: round window area, 9: extrastapedius length, 10: columella length, 11: columella volume, 12: cranial air volume, 13: interaural canal, 14: interbullar passage.

Head mass was measured after decapitation, which was performed as close to the base of the skull as possible. In 13 species, head mass was unavailable (i.e., scan data from Morphosource or no permission for decapitation), so head mass was instead calculated from skull width using the predicted values from a phylogenetic generalized least squares regression function relating skull width and head mass in 78 species of bird (R^2^ = 0.88).

# S4 Anatomical calculations

**Area calculations**

The total surface area was determined by calculating the sum of the areas of the triangles connected to the center point using Heron’s formula for the area of a triangle:

$$AREA= \surd S(S-A)(S-B)(S-C)$$

Where S equals the sum of the lengths of each triangle’s edges and A, B, C equals the individual edges of each triangle (two sides connecting the perimeter points to the center point, and one side connecting each perimeter point to the subsequent perimeter point).

**Height of umbo**

The base plane of the tympanic membrane was determined using multiple linear regression, which produced a 3D plane equation with coefficients in the form of: ax +by + cz + d = 0. The shortest distance from the umbo to the tympanic membrane base plane was calculated with the following equation:

Height of umbo = $\frac{|{ax}_{1}+{by}_{1}+{cz}_{1}+ d|}{\sqrt{a^{2}}+b^{2}+c^{2}}$

Where x_1_, y_1_, z_1_ are the coordinates of the umbo, and a, b, c are the coefficients of the 3D plane going through the base of the tympanic membrane.

**Tympanic membrane angle calculations**

The angle of the tympanic membrane conical protrusion between the line of best fit and the umbo was calculated at each tympanic membrane perimeter point. The angle (Ɵ) between each line connected to the umbo and the 3D plane was calculated with the following equation:

$Ɵ_{tympanic membrane}$ = ${sin}^{-1}\frac{|{a*u}_{1}+{b*u}_{2}+c|}{\sqrt{a^{2}+b^{2}+c^{2}}*\sqrt{{u_{1}}^{2}+{u_{2}}^{2}+{u_{3}}^{2}}}$

where u_1_, u_2_, u_3_ specify the direction vectors of the lines that connects each perimeter point to the umbo, and a, b, c are the coefficients for the 3D plane. For each specimen, this angle was computed for all 16 perimeter points and the mean angle taken.

# S5 Measurement repeatability

Measurements were done by one investigator (JNZ). Since many of the measurements required manual placement of points on the micro-CT scan data and on 3D models, they may be prone to some measurement error. To quantify such measurement error, we tested the repeatability of ear measurements using intraclass correlation coefficient for six Salvin’s prions *Pachyptila salvini* and seven rock dove *Columba livia* (using the ICCest() function of the *ICC* package [6] in R, with 3 repeated measurements for each specimen (i.e., k = 3). Mean repeatability of nine different morphological variables ranged from 0.61 to 0.99 (Fig. S4).


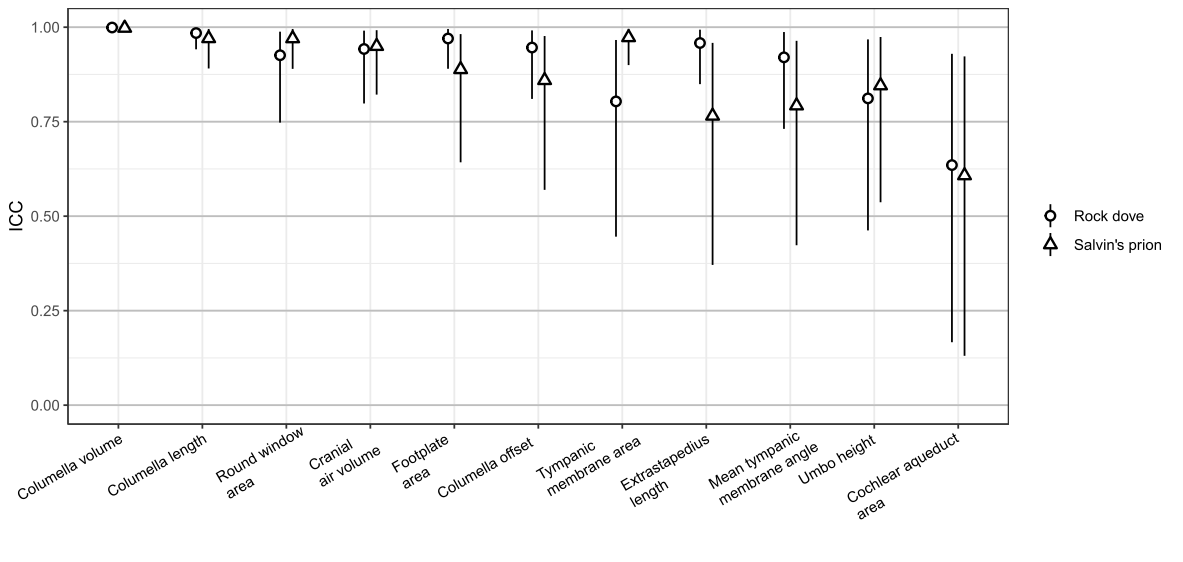


Fig. S4. Means and 95% confidence intervals of intraclass correlation coefficient for each auditory measurement, ordered from highest to lowest.

# S6 Details of ecological groupings (csv file)

For each species, the ecological group, references used for ecological group classification, dive score, maximum dive depth, average of maximum dive depths, maximum dive duration, and average of maximum dive durations are indicated, as well as the measurement technique used by each scientific article from which we extracted this information, and the reference for dive depth and reference for dive duration. A final column includes additional notes to justify classifications where literature data is limited.

# S7 Closest-related species used from birdtree.org phylogeny

| Species | Species from birdtree.org |
| --- | --- |
| *Bucorvus leadbeateri* | *Bucorvus abyssinicus* |
| *Cinnyris chalybeus* | *Aethopyga siparaja* |
| *Anthobaphes violacea* | *Nectarinia violacea* |
| *Dendropicos griseocephalus* | *Dendropicos elachus* |
| *Leucocarbo georgianus* | *Phalacrocorax atriceps* |
| *Falco rupicolus* | *Falco naumanni* |
| *Anthus cinnamomeus* | *Anthus hodgsoni* |
| *Centropus burchellii* | *Centropus superciliosus* |
| *Upupa africana* | *Upupa epops* |
| *Porphyrio madagascariensis* | *Porphyrio porphyrio* |
| *Phalacrocorax lucidus* | *Phalacrocorax carbo* |
| *Gallinula comeri* | *Gallinula tenebrosa* |
| *Gallus domesticus* | *Gallus gallus* |
| *Anas georgica georgica* | *Anas georgica* |

# S8 Bird silhouette image credits

Fig 3:

Owl - Unknown (photo), John E. McCormack, Michael G. Harvey, Brant C. Faircloth, Nicholas G. Crawford, Travis C. Glenn, Robb T. Brumfield & T. Michael Keesey. - CC3.0 - (www.phylopic.org)

- Cormorant - Ferran Sayol - no copyright - (www.phylopic.org)

*Burhinus* spp. - Aukland Museum - CC3.0 - (www.phylopic.org)

- Alcidae spp. - John James Audubon (Modified) - (no copyright) -(www.phylopic.org)

Fig S5

- Cormorant - Ferran Sayol (no copyright) (www.phylopic.org)

Dove - Dori <dori@merr.info> (source photo) and Nevit Dilmen. - CC3.0 - (www.phylopic.org)

*Burhinus* spp. - Aukland Museum - CC3.0 - (www.phylopic.org)

- *Accipiter* spp. -Ferran Sayol - (no copyright) - (www.phylopic.org)

Black guillemot - https://twitter.com/tomcadwallender/status/1274710003597877250

- Gentoo penguin -Ferran Sayol - (no copyright) - (www.phylopic.org)

Fig S6

Owl - Unknown (photo), John E. McCormack, Michael G. Harvey, Brant C. Faircloth, Nicholas G. Crawford, Travis C. Glenn, Robb T. Brumfield & T. Michael Keesey. - CC3.0 - (www.phylopic.org)

*Anhinga rufa* - Emily Willoughby - CC3.0 - (www.phylopic.org)

Fig S7

- Gentoo penguin -Ferran Sayol - (no copyright) - (www.phylopic.org)
- Cormorant - Ferran Sayol - (no copyright) - (www.phylopic.org)
- Puffin - Ferran Sayol - (no copyright) - (www.phylopic.org)

*Burhinus* spp. -Aukland Museum - CC3.0- (www.phylopic.org)

CC3.0: http://creativecommons.org/licenses/by/3.0/

# S9 Tables of phylogenetic generalised least squares regression (PGLS) summary statistics for all models

Tables S9.1 and S9.2 show the best models and details of the best models, respectively, for analyses that include terrestrial species. Tables S9.3 and S9.4 show the best models and details of the best models, respectively, for analyses that exclude terrestrial species.

| Table S9.1. Summary of AICc for all models tested comparing aquatic to terrestrial species. Best-supported model is in bold font. | | | | | | |
| --- | --- | --- | --- | --- | --- | --- |
| Anatomical category | Dependent variable | Model | Adjusted R^2^ | λ | AICc | ΔAICc |
| Impedance-matching | log(tympanic membrane area) | log(head mass) × ecology | 0.71 | 0.58 | 83 | 0 |
|  |  | **log(head mass) + ecology** | **0.7** | **0.56** | **84** | **1** |
|  |  | log(head mass) | 0.51 | 0.83 | 130 | 47 |
|  | log(footplate area) | **log(head mass) + ecology** | **0.67** | **0.75** | **44** | **0** |
|  |  | log(head mass) × ecology | 0.67 | 0.76 | 44 | 0 |
|  |  | log(head mass) | 0.62 | 0.85 | 52 | 8 |
|  | log(tympanic membrane area/footplate area) | **log(head mass)** × **ecology** | **0.27** | **0.16** | **55** | **0** |
|  |  | log(head mass) + ecology | 0.18 | 0.33 | 61 | 6 |
|  |  | log(head mass) | 0.014 | 0.51 | 79 | 24 |
|  | log(columella offset) | **log(head mass) + ecology** | **0.53** | **0.4** | **40** | **0** |
|  |  | log(head mass) × ecology | 0.54 | 0.3 | 46 | 6 |
|  |  | log(head mass) | 0.36 | 0.7 | 65 | 25 |
|  | log(umbo height) | **log(head mass) + ecology** | **0.34** | **0.43** | **100** | **0** |
|  |  | log(head mass) × ecology | 0.35 | 0.39 | 110 | 10 |
|  |  | log(head mass) | 0.13 | 0.66 | 130 | 30 |
|  | log(tympanic membrane angle) | **log(head mass) + ecology** | **0.27** | **0.011** | **55** | **0** |
|  |  | log(head mass) × ecology | 0.29 | 0.011 | 56 | 1 |
|  |  | log(head mass) | 0.046 | 0.25 | 65 | 10 |
|  | log(extrastapedius length) | **log(head mass) + ecology** | **0.59** | **0.65** | **-23** | **0** |
|  |  | log(head mass) × ecology | 0.57 | 0.68 | -17 | 6 |
|  |  | log(head mass) | 0.48 | 0.82 | -5.5 | 17.5 |
|  | log(round window area) | **log(head mass) + ecology** | **0.67** | **0.61** | **74** | **0** |
|  |  | log(head mass) × ecology | 0.67 | 0.64 | 74 | 0 |
|  |  | log(head mass) | 0.6 | 0.77 | 83 | 9 |
| Cochlear aqueduct | log(cochlear aqueduct area) | **log(head mass)** | **0.31** | **0.85** | **330** | **0** |
|  |  | log(head mass) +ecology | 0.36 | 0.8 | 330 | 0 |
|  |  | log(head mass) × ecology | 0.34 | 0.81 | 330 | 0 |
| Air volume | log(cranial air volume) | **log(head mass) + ecology** | **0.82** | **0.011** | **210** | **0** |
|  |  | log(head mass) × ecology | 0.82 | 0.064 | 220 | 10 |
|  |  | log(head mass) | 0.53 | 0.78 | 280 | 70 |
| Columella | log(columella length) | **log(head mass)** × **ecology** | **0.85** | **0.65** | **-76** | **0** |
|  |  | log(head mass) +ecology | 0.82 | 0.73 | -61 | 15 |
|  |  | log(head mass) | 0.81 | 0.75 | -57 | 19 |
|  | log(columella volume) | **log(head mass)** | **0.78** | **0.46** | **170** | **0** |
|  |  | log(head mass) +ecology | 0.8 | 0.38 | 170 | 0 |
|  |  | log(head mass) × ecology | 0.8 | 0.42 | 170 | 0 |

| Table S9.2. Model details for best-supported model comparing aquatic to terrestrial species. Terrestrial species are the reference group. Coefficients with p < 0.05 are indicated in bold font. | | | | | | |
| --- | --- | --- | --- | --- | --- | --- |
| Anatomical category | Dependent variable | Model | Coefficients | Estimate | Std. Error | p |
| Impedance-matching | log(tympanic membrane area) | log(head mass) + ecology | **(intercept)** | **1.6** | **0.15** | **<0.01** |
|  |  |  | **log(head mass)** | **0.49** | **0.03** | **<0.01** |
|  |  |  | **plunging** | **-0.63** | **0.14** | **<0.01** |
|  |  |  | **surface** | **-0.34** | **0.12** | **<0.01** |
|  |  |  | **underwater pursuit** | **-1.2** | **0.14** | **<0.01** |
|  | log(footplate area) | log(head mass) + ecology | **(intercept)** | **-1.4** | **0.15** | **<0.01** |
|  |  |  | **log(head mass)** | **0.41** | **0.026** | **<0.01** |
|  |  |  | **plunging** | **-0.27** | **0.13** | **0.039** |
|  |  |  | surface | -0.2 | 0.11 | 0.064 |
|  |  |  | **underwater pursuit** | **-0.5** | **0.12** | **<0.01** |
|  | log(tympanic membrane area/footplate area) | log(head mass) × ecology | **(intercept)** | **2.8** | **0.11** | **<0.01** |
|  |  |  | **log(head mass)** | **0.13** | **0.03** | **<0.01** |
|  |  |  | plunging | 0.0044 | 0.51 | 0.99 |
|  |  |  | surface | 0.16 | 0.23 | 0.5 |
|  |  |  | **underwater pursuit** | **1.1** | **0.45** | **0.015** |
|  |  |  | log(head mass) × plunging | -0.08 | 0.12 | 0.51 |
|  |  |  | log(head mass) × surface | -0.072 | 0.056 | 0.2 |
|  |  |  | **log(head mass)** ×  **underwater pursuit** | **-0.37** | **0.097** | **<0.01** |
|  | log(columella offset) | log(head mass) + ecology | **(intercept)** | **-0.55** | **0.11** | **<0.01** |
|  |  |  | **log(head mass)** | **0.27** | **0.024** | **<0.01** |
|  |  |  | **plunging** | **-0.31** | **0.11** | **<0.01** |
|  |  |  | **surface** | **-0.23** | **0.094** | **0.017** |
|  |  |  | **underwater pursuit** | **-0.72** | **0.11** | **<0.01** |
|  | log(umbo height) | log(head mass) + ecology | **(intercept)** | **-0.68** | **0.15** | **<0.01** |
|  |  |  | **log(head mass)** | **0.2** | **0.032** | **<0.01** |
|  |  |  | **plunging** | **-0.57** | **0.15** | **<0.01** |
|  |  |  | **surface** | **-0.41** | **0.12** | **<0.01** |
|  |  |  | **underwater pursuit** | **-0.92** | **0.14** | **<0.01** |
|  | log(tympanic membrane angle) | log(head mass) + ecology | **(intercept)** | **3.1** | **0.08** | **<0.01** |
|  |  |  | log(head mass) | -0.043 | 0.023 | 0.059 |
|  |  |  | **plunging** | **-0.29** | **0.097** | **<0.01** |
|  |  |  | **surface** | **-0.33** | **0.074** | **<0.01** |
|  |  |  | **underwater pursuit** | **-0.31** | **0.09** | **<0.01** |
|  | log(extrastapedius length) | log(head mass) + ecology | **(intercept)** | **-0.21** | **0.1** | **0.043** |
|  |  |  | **log(head mass)** | **0.26** | **0.02** | **<0.01** |
|  |  |  | **plunging** | **-0.2** | **0.095** | **0.035** |
|  |  |  | surface | -0.12 | 0.079 | 0.15 |
|  |  |  | **underwater pursuit** | **-0.5** | **0.094** | **<0.01** |
|  | log(round window area) | log(head mass) + ecology | **(intercept)** | **-0.49** | **0.15** | **<0.01** |
|  |  |  | **log(head mass)** | **0.46** | **0.029** | **<0.01** |
|  |  |  | **plunging** | **-0.56** | **0.14** | **<0.01** |
|  |  |  | **surface** | **-0.41** | **0.12** | **<0.01** |
|  |  |  | **underwater pursuit** | **-0.4** | **0.14** | **<0.01** |
|  |  |  | **(intercept)** | **-0.64** | **0.17** | **<0.01** |
| Cochlear aqueduct | log(cochlear aqueduct area) | log(head mass) | **(intercept)** | **-3.3** | **0.49** | **<0.01** |
|  |  |  | **log(head mass)** | **0.63** | **0.082** | **<0.01** |
| Air volume | log(cranial air volume) | log(head mass) + ecology | **(intercept)** | **3.2** | **0.19** | **<0.01** |
|  |  |  | **log(head mass)** | **1.1** | **0.054** | **<0.01** |
|  |  |  | plunging | -0.35 | 0.24 | 0.15 |
|  |  |  | **surface** | **-0.63** | **0.18** | **<0.01** |
|  |  |  | **underwater pursuit** | **-2.7** | **0.2** | **<0.01** |
| Columella | log(columella length) | log(head mass) × ecology | **(intercept)** | **-0.24** | **0.096** | **0.014** |
|  |  |  | **log(head mass)** | **0.36** | **0.021** | **<0.01** |
|  |  |  | plunging | -0.58 | 0.33 | 0.08 |
|  |  |  | **surface** | **-0.61** | **0.15** | **<0.01** |
|  |  |  | underwater pursuit | -0.12 | 0.23 | 0.6 |
|  |  |  | **log(head mass)** ×  **plunging** | **0.17** | **0.078** | **0.033** |
|  |  |  | **log(head mass)** ×  **surface** | **0.16** | **0.036** | **<0.01** |
|  |  |  | log(head mass) × underwater pursuit | -0.0054 | 0.054 | 0.92 |
|  | log(columella volume) | log(head mass) | **(intercept)** | **-4.6** | **0.2** | **<0.01** |
|  |  |  | **log(head mass)** | **0.84** | **0.04** | **<0.01** |

| Table S9.3. Summary of AICc for all models tested comparing aquatic species. Best-supported models are in bold font. | | | | | | |
| --- | --- | --- | --- | --- | --- | --- |
| Anatomical category | Dependent variable | Model | Adjusted R^2^ | λ | AICc | ΔAICc |
| Impedance-matching | log(tympanic membrane area) | **log(head mass) + dive score** | **0.78** | **0.0011** | **32** | **0** |
|  |  | log(head mass) × dive score | 0.78 | 0.0011 | 32 | 0 |
|  |  | log(head mass) × aquatic foraging lifestyle | 0.72 | 0.47 | 42 | 10 |
|  |  | log(head mass) + aquatic foraging lifestyle | 0.69 | 0.45 | 44 | 12 |
|  |  | log(head mass) | 0.47 | 0.74 | 67 | 35 |
|  | log(footplate area) | **log(head mass) + dive score** | **0.79** | **0.7** | **-0.62** | **0** |
|  |  | log(head mass) × dive score | 0.79 | 0.69 | 0.73 | 1.35 |
|  |  | log(head mass) + aquatic foraging lifestyle | 0.77 | 0.71 | 4.1 | 4.72 |
|  |  | log(head mass) × aquatic foraging lifestyle | 0.78 | 0.7 | 6.6 | 7.22 |
|  |  | log(head mass) | 0.73 | 0.78 | 9.9 | 10.52 |
|  | log(tympanic membrane area/footplate area) | **log(head mass) + dive score** | **0.22** | **0.045** | **42** | **0** |
|  |  | log(head mass) × dive score | 0.24 | 0.0011 | 43 | 1 |
|  |  | log(head mass) × aquatic foraging lifestyle | 0.18 | 0.2 | 47 | 5 |
|  |  | log(head mass) + aquatic foraging lifestyle | 0.12 | 0.36 | 48 | 6 |
|  |  | log(head mass) | -0.018 | 0.46 | 53 | 11 |
|  | log(columella offset) | **log(head mass) + dive score** | **0.4** | **0.63** | **36** | **0** |
|  |  | log(head mass) + aquatic foraging lifestyle | 0.4 | 0.43 | 38 | 2 |
|  |  | log(head mass) × dive score | 0.38 | 0.63 | 39 | 3 |
|  |  | log(head mass) × aquatic foraging lifestyle | 0.39 | 0.38 | 43 | 7 |
|  |  | log(head mass) | 0.27 | 0.6 | 46 | 10 |
|  | log(umbo height) | **log(head mass) + dive score** | **0.13** | **0.0011** | **61** | **0** |
|  |  | log(head mass) × dive score | 0.12 | 0.0011 | 63 | 2 |
|  |  | log(head mass) + aquatic foraging lifestyle | 0.17 | 0.35 | 64 | 3 |
|  |  | log(head mass) | 0.061 | 0.27 | 68 | 7 |
|  |  | log(head mass) × aquatic foraging lifestyle | 0.15 | 0.35 | 68 | 7 |
|  | log(tympanic membrane angle) | **log(head mass)** | **0.024** | **0.0011** | **52** | **0** |
|  |  | log(head mass) + dive score | 0.016 | 0.0011 | 52 | 0 |
|  |  | log(head mass) × dive score | 0.0089 | 0.0011 | 54 | 2 |
|  |  | log(head mass) + aquatic foraging lifestyle | -0.011 | 0.0011 | 56 | 4 |
|  |  | log(head mass) × aquatic foraging lifestyle | 0.0032 | 0.0011 | 58 | 6 |
|  | log(extrastap-edius length) | **log(head mass) + aquatic foraging lifestyle** | **0.5** | **0.75** | **4.6** | **0** |
|  |  | log(head mass) × aquatic foraging lifestyle | 0.49 | 0.79 | 8.2 | 3.6 |
|  |  | log(head mass) + dive score | 0.43 | 0.8 | 10 | 5.4 |
|  |  | log(head mass) × dive score | 0.44 | 0.83 | 11 | 6.4 |
|  |  | log(head mass) | 0.39 | 0.83 | 12 | 7.4 |
|  | log(round window area) | **log(head mass)** | **0.62** | **0.51** | **26** | **0** |
|  |  | log(head mass) + aquatic foraging lifestyle | 0.65 | 0.38 | 28 | 2 |
|  |  | log(head mass) + dive score | 0.62 | 0.48 | 29 | 3 |
|  |  | log(head mass) × aquatic foraging lifestyle | 0.75 | 0.0011 | 30 | 4 |
|  |  | log(head mass) × dive score | 0.6 | 0.53 | 31 | 5 |
| Cochlear aqueduct | log(cochlear aqueduct area) | **log(head mass) + dive score** | **0.55** | **0.92** | **97** | **0** |
|  |  | log(head mass) × dive score | 0.56 | 0.92 | 97 | 0 |
|  |  | log(head mass) + aquatic foraging lifestyle | 0.54 | 0.93 | 99 | 2 |
|  |  | log(head mass) | 0.52 | 0.93 | 100 | 3 |
|  |  | log(head mass) × aquatic foraging lifestyle | 0.53 | 0.93 | 100 | 3 |
| Air volume | log(cranial air volume) | **log(head mass) + aquatic foraging lifestyle** | **0.78** | **0.2** | **110** | **0** |
|  |  | log(head mass) × aquatic foraging lifestyle | 0.77 | 0.14 | 120 | 10 |
|  |  | log(head mass) + dive score | 0.7 | 0.0011 | 130 | 20 |
|  |  | log(head mass) × dive score | 0.69 | 0.0011 | 130 | 20 |
|  |  | log(head mass) | 0.47 | 0.83 | 150 | 40 |
| Columella | log(columella length) | **log(head mass)** × **aquatic foraging lifestyle** | **0.87** | **0.8** | **-39** | **0** |
|  |  | log(head mass) + aquatic foraging lifestyle | 0.86 | 0.81 | -35 | 4 |
|  |  | log(head mass) + dive score | 0.84 | 0.83 | -30 | 9 |
|  |  | log(head mass) × dive score | 0.85 | 0.81 | -30 | 9 |
|  |  | log(head mass) | 0.83 | 0.85 | -29 | 10 |
|  | log(columella volume) | **log(head mass) + aquatic foraging lifestyle** | **0.85** | **0.0011** | **69** | **0** |
|  |  | log(head mass) | 0.83 | 0.0011 | 72 | 3 |
|  |  | log(head mass) × aquatic foraging lifestyle | 0.85 | 0.0011 | 72 | 3 |
|  |  | log(head mass) + dive score | 0.83 | 0.0011 | 73 | 4 |
|  |  | log(head mass) × dive score | 0.83 | 0.0011 | 75 | 6 |

| Table 9.4. Model details for best-supported model comparing among aquatic species. P < 0.05 are indicated in bold. | | | | | | |
| --- | --- | --- | --- | --- | --- | --- |
| Anatomical category | Dependent variable | Model | Coefficients | Estimate | Std. Error | p |
| Impedance-matching | log(tympanic membrane area) | log(head mass) + dive score | **(intercept)** | **0.83** | **0.18** | **<0.01** |
|  |  |  | **log(head mass)** | **0.57** | **0.043** | **<0.01** |
|  |  |  | **divescore** | **-0.29** | **0.032** | **<0.01** |
|  | log(footplate area) | log(head mass) + dive score | **(intercept)** | **-2.1** | **0.18** | **<0.01** |
|  |  |  | **log(head mass)** | **0.51** | **0.035** | **<0.01** |
|  |  |  | **divescore** | **-0.11** | **0.03** | **<0.01** |
|  | log(tympanic membrane area/footplate area) | log(head mass) + dive score | **(intercept)** | **3.1** | **0.2** | **<0.01** |
|  |  |  | log(head mass) | 0.04 | 0.047 | 0.4 |
|  |  |  | **divescore** | **-0.15** | **0.036** | **<0.01** |
|  | log(columella offset) | log(head mass) + dive score | **(intercept)** | **-0.85** | **0.25** | **<0.01** |
|  |  |  | **log(head mass)** | **0.29** | **0.05** | **<0.01** |
|  |  |  | **divescore** | **-0.14** | **0.042** | **<0.01** |
|  | log(umbo height) | log(head mass) + dive score | **(intercept)** | **-1.1** | **0.24** | **<0.01** |
|  |  |  | **log(head mass)** | **0.16** | **0.057** | **<0.01** |
|  |  |  | **divescore** | **-0.1** | **0.043** | **0.02** |
|  | log(tympanic membrane angle) | log(head mass) | **(intercept)** | **2.9** | **0.22** | **<0.01** |
|  |  |  | log(head mass) | -0.074 | 0.049 | 0.13 |
|  | log(extrastapedius length) | log(head mass) + aquatic foraging style | (intercept) | -0.37 | 0.2 | 0.067 |
|  |  |  | **log(head mass)** | **0.26** | **0.037** | **<0.01** |
|  |  |  | plunging | -0.087 | 0.096 | 0.37 |
|  |  |  | **underwater pursuit** | **-0.39** | **0.11** | **<0.01** |
|  | log(round window area) | log(head mass) | **(intercept)** | **-0.75** | **0.22** | **<0.01** |
|  |  |  | **log(head mass)** | **0.41** | **0.044** | **<0.01** |
| Cochlear aqueduct | log(cochlear aqueduct area) | log(head mass) + dive score | **(intercept)** | **-2.6** | **0.48** | **<0.01** |
|  |  |  | **log(head mass)** | **0.61** | **0.084** | **<0.01** |
|  |  |  | **dive score** | **0.15** | **0.076** | **0.046** |
| Airvolume | log(cranial air volume) | log(head mass) + aquatic foraging style | **(intercept)** | **1.9** | **0.56** | **<0.01** |
|  |  |  | **log(head mass)** | **1.3** | **0.12** | **<0.01** |
|  |  |  | plunging | 0.19 | 0.3 | 0.52 |
|  |  |  | **underwater pursuit** | **-2.2** | **0.28** | **<0.01** |
| Columella | log(columella length) | log(head mass) × aquatic foraging style | **(intercept)** | **-0.79** | **0.14** | **<0.01** |
|  |  |  | **log(head mass)** | **0.5** | **0.029** | **<0.01** |
|  |  |  | plunging | 0.0025 | 0.33 | 0.99 |
|  |  |  | underwater pursuit | 0.48 | 0.24 | 0.052 |
|  |  |  | log(head mass) × plunging | 0.018 | 0.079 | 0.83 |
|  |  |  | **log(head mass) × underwater pursuit** | **-0.16** | **0.057** | **<0.01** |
|  | log(columella volume) | log(head mass) + aquatic foraging style | **(intercept)** | **-5.2** | **0.23** | **<0.01** |
|  |  |  | **log(head mass)** | **0.94** | **0.052** | **<0.01** |
|  |  |  | plunging | 0.22 | 0.15 | 0.14 |
|  |  |  | underwater pursuit | -0.21 | 0.13 | 0.11 |

# S10 Examples of anatomical differences in micro-CT scans

The examples below highlight anatomical differences between aquatic and terrestrial birds via micro-CT slices and 3D renderings. These include differences in the columella footplate and tympanic membrane sizes (Fig S5), tympanic membrane angle and the offset of the columella from the center of the tympanic membrane base (Fig S6), cranial air volume and connectivity (Fig S7) and large cochlear aqueduct (Fig S8).


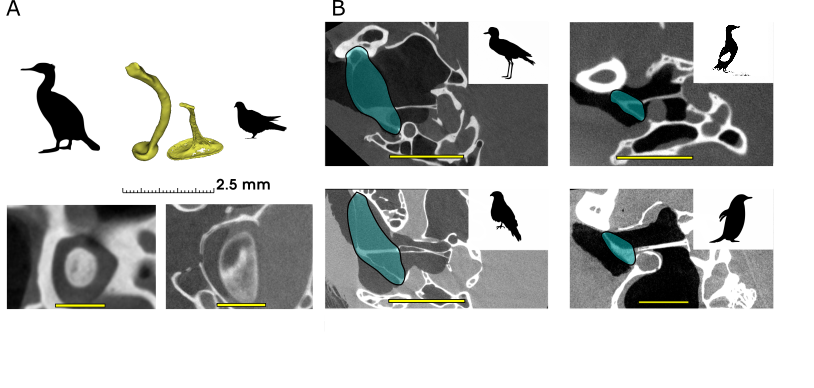


Fig. S5. (A) Differences in columella footplate area are illustrated by comparing the columellas of the Cape cormorant *Phalacrocorax capensis* (left), and that of the much smaller, red-eyed dove *Streptopelia semitorquata* (right), which has a much larger footplate. Bottom images are micro-CT slices fit to the plane of respective footplates. Yellow bars in (A) indicate 1 mm. (B) Variation in tympanic membrane size is shown with micro-CT slices aligned through the shaft of the columella of the spotted thick-knee *Burhinus capensis* and the black guillemot *Cepphus grylle*, both members of Charadriformes (top) and two unrelated species, the black sparrowhawk *Accipiter melanoleucus* and the gentoo penguin *Pygoscelis papua* (bottom). Both the tympanic membrane and extracolumella are highlighted in blue. Yellow bars in (B) indicate 5 mm.


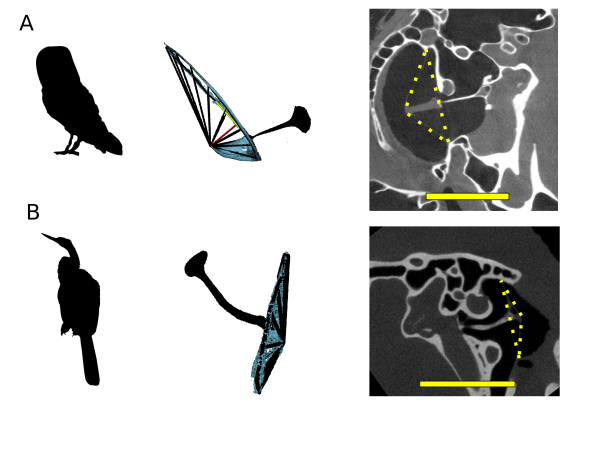


Fig. S6. Comparison between the barn owl *Tyto alba* and the African darter *Anhinga rufa*, illustrating differences related to conical protrusion of the eardrum, columella offset, and extrastapedius length. 3D models (middle) and micro-CT slices through the plane of the columella shaft (right) are shown. Differences in the angle of the eardrum protrusion highlighted with yellow dotted lines. Yellow bars indicate 5 mm.


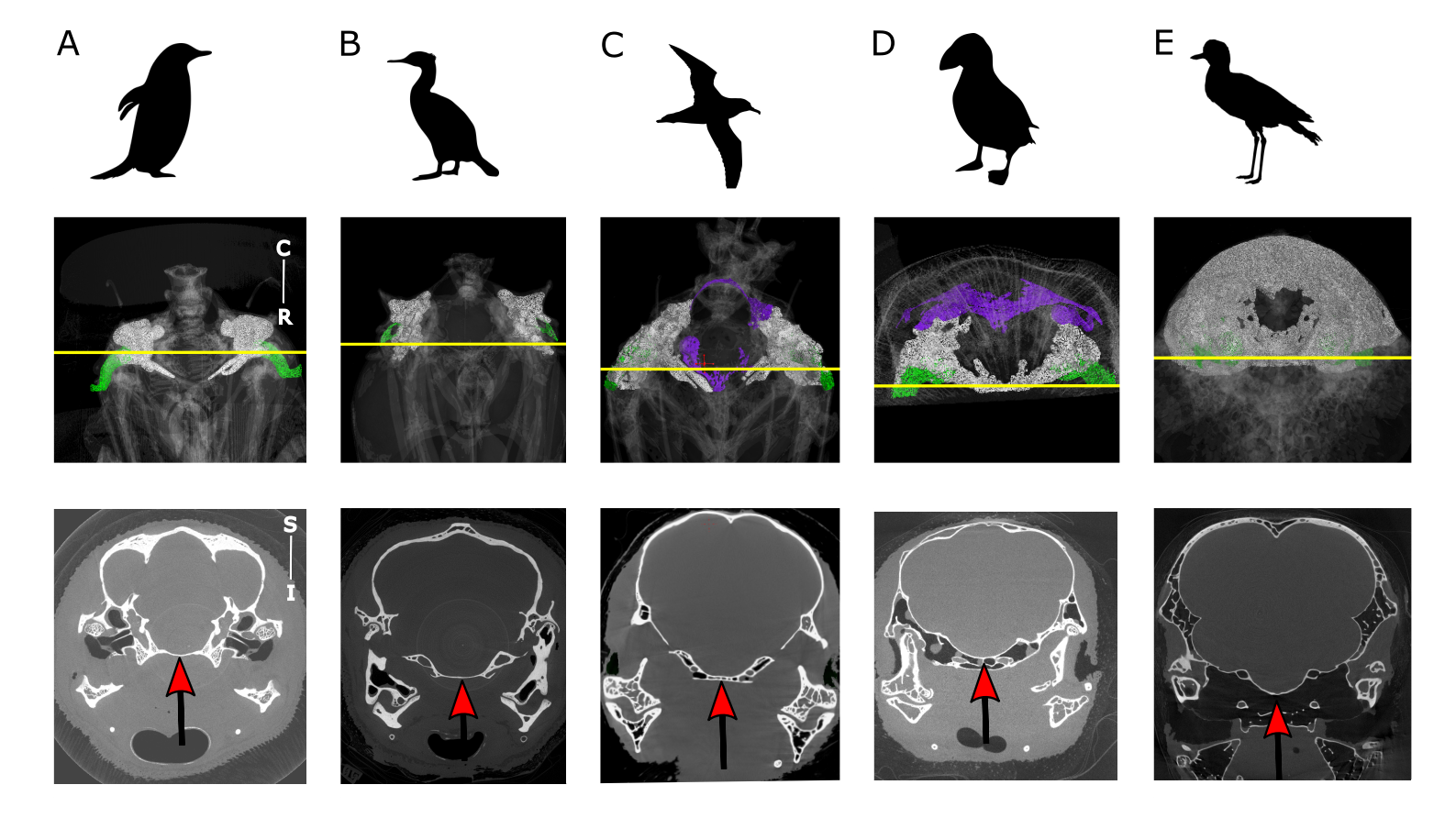


Fig. S7. Variation in air volume is demonstrated in four diving species and a terrestrial species. This includes a 3D rendering from the top view (C = caudal, R = rostral) (white = air behind eardrum, green = ear canal, purple = soft tissue), and a micro-CT slice through the frontal plane (S= superior, I = inferior). Yellow line indicates position of frontal slice. Gentoo penguin *Pygoscelis papua* (A) and South Georgia shag *Leucocarbo georgianus* (B) show a complete absence of opening in the bone for the interaural canal and interbullar passage. Sooty shearwater *Ardenna grisea* (C) has narrow interaural canal and interbullar passage, both filled with soft tissue. Atlantic puffin *Fratercula arctica* (D) shows well-developed interaural canal. Spotted thick-knee *Burhinus capensis* (E) shows a well-developed interaural canal and interbullar passage. Red arrows highlight the presence or absence of the interaural canal in the micro-CT slice.


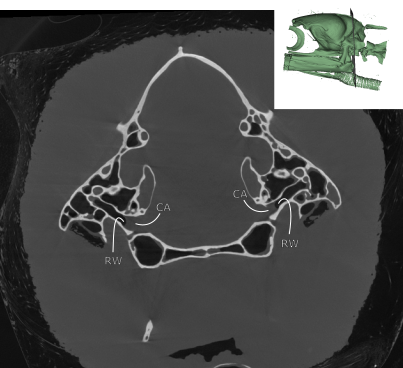


Fig. S8. Micro-CT slice through the frontal plane of a Cape cormorant *Phalacrocorax capensis* head, demonstrating the large cochlear aqueduct connecting the ear to the cranial cavity. RW = round window, CA = cochlear aqueduct.

# S11 Dive depth and dive duration correlations


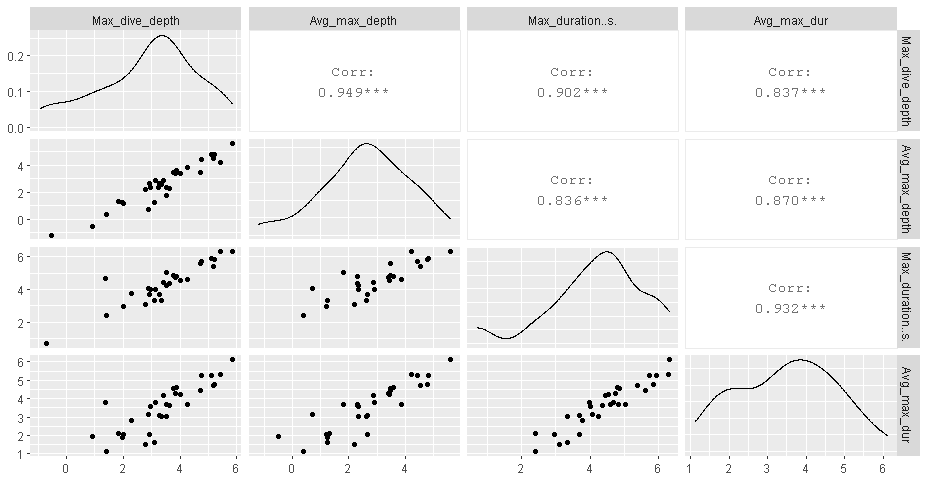


Fig. S9 Bi-variate correlations between maximum dive depth, average maximum depth, maximum duration, and average maximum duration.

# S12 Narrow ear canal of king penguin


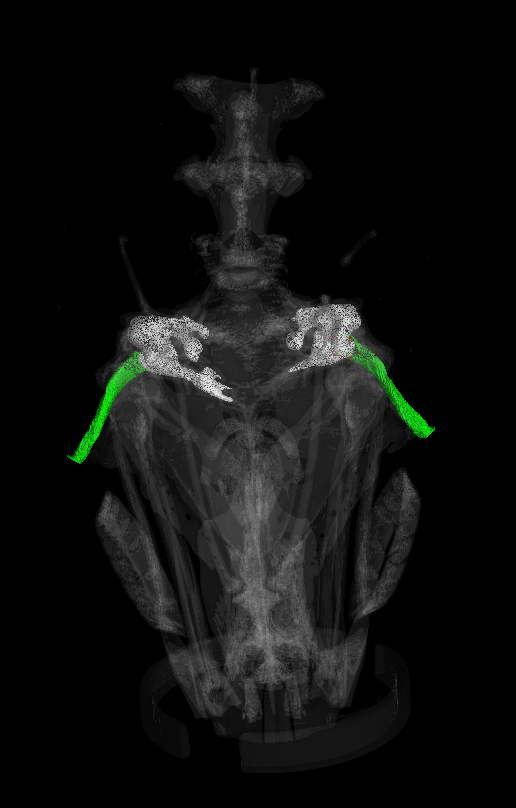


Fig. S10. Three dimensional rendering of the long narrow ear canal (green) and middle ear air cavities (white) of the king penguin *Aptenodytes patagonicus*.

# References

1. Schwartzkopff J. 1956 Die Größenverhältnisse von Trommelfell Columella-Fußplatte und Schnecke bei Vögeln verschiedenen gewichts. *Z. Für Morphol. Ökol. Tiere* **45**, 365–378. (doi:10.1007/BF00407703)

2. Kartaschew N, Iljitschwe WD. 1964 Uber das Gehörorgan der Alkenvogel. J Ornithol 105, 113–136.

3. Starck JM. 1995 Results. In *Comparative Anatomy of the External and Middle Ear of Palaeognathous Birds*, pp. 21-111. Springer, Berlin, Heidelberg. (doi:10.1007/978-3-642-79592-3_1)

4. Adams DC, Collyer ML, Kaliontzopoulou A. 2020 *Geomorph: Software for geometric morphometric analyses. R package version 3.2.1.* See https://cran.r-project.org/package=geomorph.

5. Cignoni P, Callieri M, Corsini M, Dellepiane M, Ganovelli F, Ranzuglia G. 2008 Meshlab: an open-source mesh processing tool. In *Eurographics Italian chapter conference*, pp. 129–136.

6. Wolak ME, Fairbairn DJ, Paulsen YR. 2012 Guidelines for estimating repeatability. *Methods Ecol. Evol.* **3**, 129–137. (doi:10.1111/j.2041-210X.2011.00125.x)
